# Mycoplasmas are no exception to extracellular vesicles release: Revisiting old concepts

**DOI:** 10.1371/journal.pone.0208160

**Published:** 2018-11-28

**Authors:** Patrice Gaurivaud, Sarah Ganter, Alexandre Villard, Lucia Manso-Silvan, Didier Chevret, Christelle Boulé, Véronique Monnet, Florence Tardy

**Affiliations:** 1 Université de Lyon, Anses, Laboratoire de Lyon, UMR Mycoplasmoses des Ruminants, Lyon, France; 2 Université de Lyon, VetAgro Sup, UMR Mycoplasmoses des Ruminants, Marcy-L’étoile, France; 3 CIRAD, UMR ASTRE, Montpellier, France; 4 INRA, UMR ASTRE, Montpellier, France; 5 PAPPSO, Micalis Institute, INRA, AgroParisTech, Université Paris-Saclay, Jouy-en-Josas, France; 6 Université Claude Bernard Lyon 1, Centre Technologique des Microstructures, Service « Etudes à façon » EZUS Lyon, Villeurbanne, France; University of North Dakota School of Medicine and Health Sciences, UNITED STATES

## Abstract

Release of extracellular vesicles (EV) by Gram-negative and positive bacteria is being frequently reported. EV are nano-sized, membrane-derived, non-self-replicating, spherical structures shed into the extracellular environment that could play a role in bacteria-host interactions. Evidence of EV production in bacteria belonging to the class *Mollicutes*, which are wall-less, is mainly restricted to the genus *Acholeplasma* and is scanty for the *Mycoplasma* genus that comprises major human and animal pathogens. Here EV release by six *Mycoplasma* (sub)species of clinical importance was investigated. EV were obtained under nutritional stress conditions, purified by ultracentrifugation and observed by electron microscopy. The membrane proteins of EV from three different species were further identified by mass spectrometry as a preliminary approach to determining their potential role in host-pathogen interactions. EV were shown to be released by all six (sub)species although their quantities and sizes (30–220 nm) were very variable. EV purification was complicated by the minute size of viable mycoplasmal cells. The proteins of EV-membranes from three (sub)species included major components of host-pathogen interactions, suggesting that EV could contribute to make the host-pathogen interplay more complex. The process behind EV release has yet to be deciphered, although several observations demonstrated their active release from the plasma membrane of living cells. This work shed new light on old concepts of “elementary bodies” and “not-cell bound antigens”.

## Introduction

Extracellular vesicles (EV) are nano-sized, membrane-derived, non-self-replicating, spherical structures shed into the extracellular environment by both eukaryotic and prokaryotic cells [1]. Most prokaryotic EV studied since the earliest observations in the 1960s [2] are Gram-negative outer membrane vesicles [3]. Their composition, biogenesis and role in bacterial virulence and cross-talk with eukaryotic cells have been deciphered in various bacterial models [4]. In contrast, EV from Gram-positive bacteria have been poorly characterized up to now even though they were first observed by electron microscopy in a *Bacillus* culture as early as 1990 [5]. The first characterization of EV released by a Gram-positive bacteria came from the seminal study of *Staphylococcus aureus* in 2009 [6] and, since then, EV have been described in several Gram-positive genera, such as *Bacillus*, *Streptomyces*, *Listeria*, *Clostridium* and *Streptococcus* [7–11]. In the absence of an outer membrane, Gram-positive EV are formed from the cytoplasmic membrane. Hence the thick peptidoglycan cell wall is certainly a physical barrier that could hinder their release and explain the low yield of EV production from Gram-positive bacteria *in vitro* [12].

Within the Gram-positive phylum, bacteria belonging to the class *Mollicutes* hold a special place as they are wall-less, minute-sized cells limited only by a cytoplasmic membrane. They evolved from a common Gram-positive ancestor with a low G+C content and are phylogenetically close to *Clostridium* spp. [13]. The genetic relatedness with the EV-producing *Clostridium* genus [7] and the lack of a cell wall physical barrier are two reasons why we could expect EV production from *Mollicutes*. This was indeed demonstrated by Chernov and collaborators in 2011 using *Acholeplasma* (*A*.) *laidlawii* as a model [14]. *A*. *laidlawii* is a filament-shaped mollicute (0.5 μm wide, 2.0 μm long [15]), widespread in nature. The EV observed by electron microscopy from a culture grown under starvation conditions were 70 to 120 nm in length [14] and contained DNA, RNA as well as several proteins some of which could be involved in *Acholeplasma*-plant interactions [14, 16]. The same group also observed vesicles from *Mycoplasma* (*M*.) *gallisepticum* by atomic force microscopy but did not characterize them further [14].

Although they belong to the same *Mollicutes* class, bacteria from the genus *Mycoplasma* differ from those of the genus *Acholeplasma*. They are spherical, 0.3 to 1.0 μm in diameter [15] and carry a smaller genome (580–1350 kpb for mycoplasmas versus 1500–1650 kpb for acholeplasmas). Furthermore, unlike acholeplasmas, their membrane is rich in cholesterol that has to be provided by their growth environment as they are not able to synthesize it. Finally, whereas *Acholeplasma* species are most often regarded as opportunists, *Mycoplasma* species include several important pathogens for humans and animals. Hence, their capacity to produce EV might be of interest in terms of host-pathogen interactions.

The aim in the present work was to investigate EV release by several *Mycoplasma* species. A nutritional stress was applied to stimulate EV production and the general method recommended for EV purification was used and validated on the *A*. *laidlawii* model. Six *Mycoplasma* (sub)species, including pathogens of both human and animal origin, were shown to produce EV under nutritional stress. In vitro tests showed that EV are produced by living mycoplasma cells and are neither the result of dead cell lysis nor of aberrant cell division process. Proteomic analyses further revealed that EV-membranes contained proteins potentially involved in host-pathogen interactions.

## Material and methods

### Strains and culture conditions

The *Acholeplasma* and *Mycoplasma* species and strains used in this study are listed in Table 1. Of note for *M*. *mycoides* subsp. *mycoides* strain Afadé, only the TR-variant was used because i) it has no polysaccharide capsule, which is expected to facilitate EV release and ii) it expresses constitutively the glucose permease (MSC_0860) recognized by the 3f3 monoclonal antibody, which is used as a marker (see hereafter) [17]. They were propagated at 37°C under 5% CO_2_ in complete mycoplasma medium (PPLO) composed of a base (mycoplasma broth base 1.4% (w/v), tryptose 1.0% (w/v), yeast extract 0.3% (w/v), glucose 0.1% (w/v)), adjusted to pH 7.6, with addition of 30% supplement (horse serum 48% (v/v), pig serum 16% (v/v), fresh yeast extract 32% (v/v), cysteine 0.1% (w/v), NAD 0.1% (w/v), thallium acetate 0.3% (w/v)). For EV production the PPLO broth was modified (m-PPLO) by reducing the supplement fraction to 10% and adding 20% of 1X MEM α nucleoside (ThermoFisher) to the final medium to ensure substantial serum depletion (from 19% to 6%) without modifying the osmotic pressure. The supplement used for m-PPLO preparation was previously ultracentrifuged at 100 000g for 15h at 8°C in order to deplete potential exogenous eukaryotic extracellular vesicles. The PPLO and m-PPLO media were supplemented with amoxicillin 1g/l. Viable cell concentrations (cfu/ml) were determined by plating serial dilutions of liquid cultures onto PPLO agar plates. Each count was done in triplicate.

**Table 1:** List and main characteristics of strains used for EV preparation. Follow-up and quality control of EV production.

Phylogenetic groups	Species/subspecies	Strains	Main hosts	Clinical signs	CFU/ml in m-PPLO^a^			EV preparation	
					0h	24h	48h or 96h	Density^b^	Cell contamination^c^
**Phytoplasma, Acholeplasma**	*A*. *laidlawii*	PG8^T^	Vertebrate and invertebrate	none	2.1+/-0.6x10^9^	1.0+/-0.8x10^9^	(96h) 3.5+/-2.4x10^7^	+	56/1.8x10^10^
**Spiroplasma**	*M*. *mycoides* subsp. *mycoides*	Afadé^d^	Cattle	Contagious bovine pleuropneumonia	9.8+/-1.7x10^8^	3.2+/-0.8x10^9^	(96h) 4.9+/-4.3x10^7^	++	0/2.5x10^10^
	*M*. *mycoides* subsp. *capri*	PG3^T^	Goat	Contagious agalactia	8.4+/-0.4x10^8^	4.8+/-0.9x10^9^	(48h) 3.4+/-0.0x10^8^ (96h) 9.5+/-2.2x10^4^	+/-	1224/3.4x10^10^
	*M*. *capricolum* subsp. *capricolum*	L15937	Goat	Contagious agalactia	1.6+/-0.2x10^9^	2.3+/-0.4x10^8^	(48h) 8.6+/-0.3x10^6^ (96h) 1.1+/-0.6x10^5^	+	0/8.6x10^8^
**Hominis**	*M*. *agalactiae*	L14628	Alpine ibex	Pneumonia	3.9+/-0.2x10^8^	3.5+/-0.8x10^9^	(96h) 5.6+/-0.3x10^8^	+	UC/5.6x10^10^
		5632	Goat	Contagious agalactia	1.5+/-0.2x10^9^	2.6+/-0.4x10^8^	(96h) 2.2+/-0.0x10^8^	+	624/1.1x10^11^
	*M*. *fermentans*	PG18^T^	Human	Found associated with various diseases and clinical syndromes	2.1+/-0.3x10^8^	4.4+/-0.6x10^8^	(96h) 2.0+/-0.0x10^6^	+++	0/2.0x10^8^
	*M*. *bovis*	L15762	Cattle	Bovine respiratory complex disease	9.3+/-1.5x10^8^	2.0+/-0.5x10^9^	(96h) 1.9+/-0.1x10^6^	+	1068/1.9x10^8^

^a^ Data correspond to mean +/- standard deviation of 3 cell counts, except for *M*. *mycoides* subsp. *mycoides* for which data correspond to mean +/- standard deviation of the three batches *i*.*e*. 9 cell counts.

^b^ EV density in electron micrographs was estimated using the following scale: +/-, counts <1 EV/ 5μm^2^; +, 1–10 EV/ 5μm^2^; ++, 10–100 EV/ 5μm^2^; +++ >100 EV/ 5μm^2^.

^c^ Total number of CFU in EV extract / number of CFU at 48h or 96h (CFU/ml x volume of m-PPLO medium used for EV preparation)

^d^ the non-capsulated variant TR was used in this study (see Material and methods)

UC, uncountable; T, type strain.

*M*. *mycoides* subsp. *mycoides* strain Afadé variant TR was further used as a model to investigate the link between cell viability and EV production. Firstly, mycoplasma cells from a 24h-culture in m-PPLO medium (10^9^ cfu/ml) were harvested (12000 g, 20 min, 18°C), resuspended in PBS and one half of the cells were incubated at 60°C for 1 hour while the other was incubated at room temperature for 1h. Cells from both batches were inoculated in m-PPLO medium and further incubated for 72h at 37°C before vesicles extraction and cell counts. In the second experiment, a 24h-culture (10^8^ cfu/ml) was divided into two batches. One of the batches was supplemented with 200μg gentamicin/ml (a concentration known to be mycoplasmacidal [18]). A further 72h incubation at 37°C was performed before vesicles purification (see herafeter). The relative concentration of vesicles was estimated by electron microscopy and/or 3f3 immunobinding [19].

### Isolation of extracellular vesicles

*Acholeplasma* and *Mycoplasma* strains were first cultivated in PPLO broth for 48h at 37°C, 5% CO_2_. Cells were harvested by centrifugation (12000g, 30min, 20°C), suspended in m-PPLO broth and further incubated at 37°C for 4 days, except for *M*. *mycoides* subsp. *capri* and *M*. *capricolum* subsp. *capricolum*, which were incubated for 2 days. Cells were removed by centrifugation at 14000g for 1h at 4°C and the supernatants were sterilized by filtering through 0.22 μm PVDF membranes. The filtrates were then concentrated 4-fold with Vivacell 70 MWCO 100 kDa (Sartorius). EV were collected after ultracentrifugation at 100 000g for 90 min at 8°C, then washed and suspended in 100 μl sterile PBS (10 mM phosphate, 150 mM NaCl, pH 7.8). All EV preparations were stored at -80°C and all further manipulations were carried out at 4°C. The absence of viable mycoplasma cells in EV preparations was ascertained by plating 25 μl (i.e. ¼) of the EV preparation onto PPLO agar plates.

### Transmission electron microscopy (TEM) observation

The 200-mesh copper grids coated with Formvar-carbon were glow-discharged, and 5-μL samples were directly loaded onto the grids for two minutes before absorbing the liquid excess. The samples were then negatively stained by floating for 30 seconds on a drop of 1.0% (w/v) sodium silicotungstate. After air drying, images were acquired using a Philips CM120 TEM operated at 80 kV. EV diameter was determined using ImageJ software [20]. The mean EV density per (sub)species was estimated from the number of vesicles observed in ten 5 μm^2^ squares.

### EV membrane proteomics

Since EV-protein quantification was biased by the presence of proteinaceous contaminants that co-purify with EV despite PBS washing (as observed in electron micrographs, see for example S2 Fig), EV-membrane proteins were extracted from 50μl of each EV preparation batch using Triton X-114 phase partitioning [21]. Proteins in the detergent phase were precipitated with methanol and chloroform, resuspended in Laemmli buffer and boiled for 5 min. Samples were electrophoresed on a miniprotean Any kd TGX gel (Bio-Rad) at 150 V for 5 min. The gels were stained with blue-safe staining from Bio-Rad according to the supplier’s instructions. Gel bands were excised and washed with DTT and iodoacetamide. In-gel tryptic digestion was performed overnight with 100 ng of trypsin (sequencing grade from Promega) in bicarbonate buffer 6h at 37°C. Peptides were extracted with 5% formic acid in water/acetonitrile (v/v), dried and suspended in 25μl of 0.1% formic acid (v/v) and 2% acetonitrile (v/v). LC MS/MS analyses were performed using an Ultimate 3000 RSLC system (Dionex) connected to a LTQ orbitrap mass spectrophotometer (ThermoFisher) by a nanoelectrospray ion source.

All MS/MS spectra were searched against Uniprot databases (*Mycoplasma (M*.*) mycoides* subsp. *mycoides* PG1^T^ (Uniprot proteome identifier UP000001016, version 2013/10/11, 978 entries), *M*. *agalactiae* PG2^T^ (Uniprot proteome identifier UP000007065, 11/04/2017, 726 entries) and *M*. *fermentans* PG18^T^ (Uniprot proteome identifier UP000006810, 13/05/2017, 1091 entries) by the X!TandemPipeline (open source software developed by PAPPSO, version 3.4.3 [22]). The identified proteins were filtered using E-values <10^−4^ for proteins and <0.01 for peptides with a minimum of 2 peptides. A protein abundance index (PAI) was calculated for each protein in each sample using a relative quantification method, based on spectral counts. PAI, which reflects the relative abundance of the different proteins in each sample, is defined as the number of fragmentation spectra assigned with a significant score divided by the number of observable peptides per protein [23]. Membrane proteins, *i*.*e*. lipoproteins and transmembrane proteins were identified using Uniprot annotations and TMHMM2.0 prediction [24]. Other proteins were identified as either cytoplasmic or membrane-bound in reference to functional data but in the absence of characteristic structural features.

## Results & discussion

### Validation of our in-house experimental procedure for EV production

**Fig 1:**
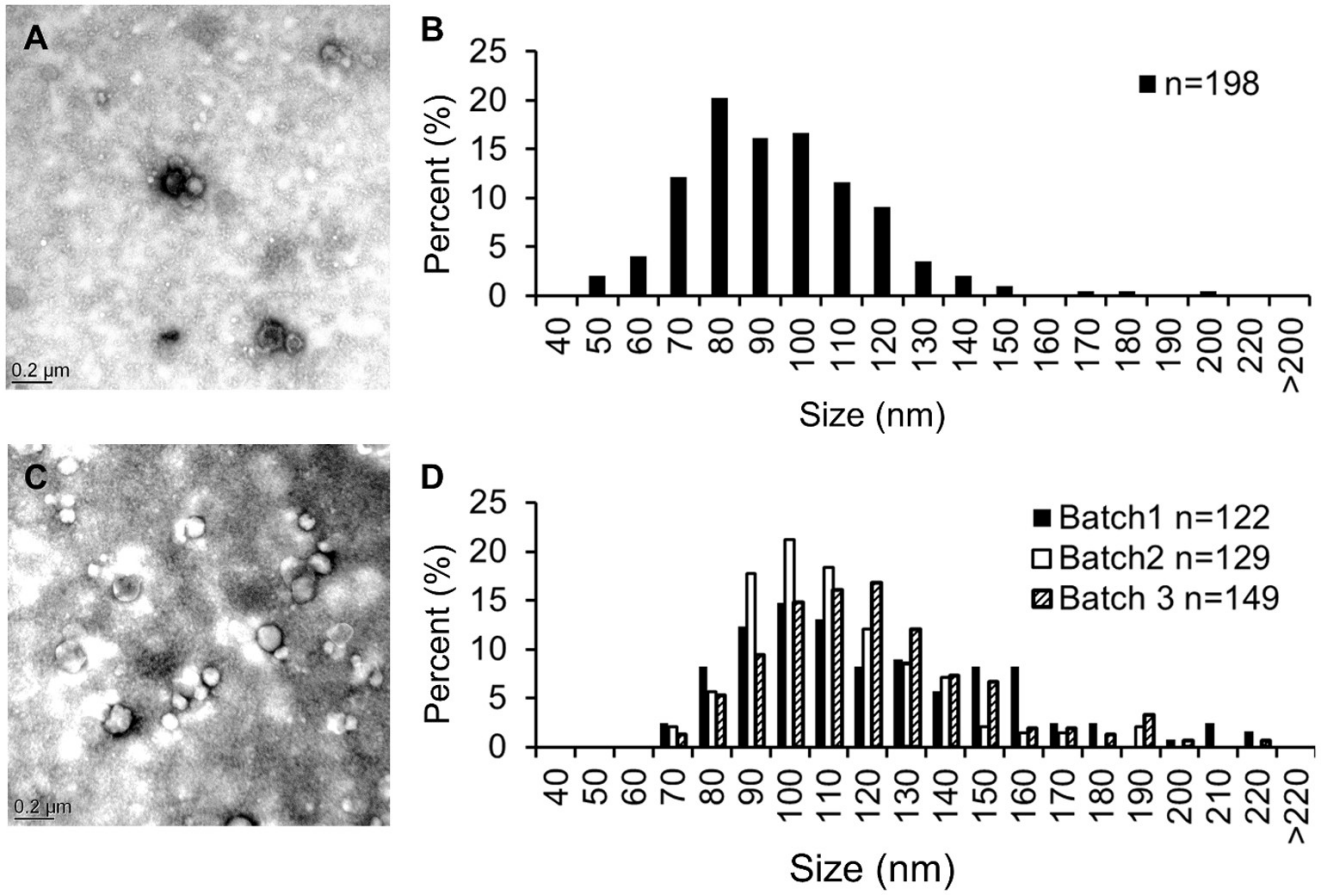
Electron micrographs (A, C) and size distributions (B, D) of negatively-stained EV purified from *Acholeplasma laidlawii* PG8^T^ (A, B) and *Mycoplasma mycoides* subsp. *mycoides* Afadé (C, D). EV diameters were estimated using Image J on n = 198 vesicles from *A*. *laidlawii* PG8^T^ and n = 122, 129 and 149 vesicles from *M*. *mycoides* subsp. *mycoides* Afadé, corresponding to three production batches.

The nutritional stress applied to mycoplasma cultures involved growth in m-PPLO medium, which was partially-depleted in animal serum and fresh yeast extract to reduce the quantities of cholesterol, lipids, peptides, vitamins and nucleotides available for cell division. *A*. *laidlawii* strain PG8^T^ was used as a control to validate our capacity to induce EV formation and perform purification. Cells were first cultivated in complete mycoplasma medium (PPLO) until the end of the log phase and then transferred to m-PPLO at a concentration of 10^8^–10^9^ cfu/ml for further incubation (Table 1). The titer of viable *Acholeplasma* cells remained steady for at least 24h and dropped to 10^7^ cfu/ml at 96h (Table 1). EV were purified from the 96h-supernatant by ultracentrifugation and observed by TEM. The sizes ranged from 50 to 150 nm, with a few elements >150nm (Fig 1), consistent with the observations by Chernov and collaborators [14], and an estimated density of 5 EV/ 5μm^2^ field. The same experimental conditions were then tested on *M*. *mycoides* subsp. *mycoides* (*Mmm*) strain Afadé used as a representative of the *Mycoplasma* genus. Three different EV batches were prepared to assess the reproducibility of the technique. The viability of *Mmm* strain Afadé in m-PPLO was similar to that of *A*. *laidlawii* strain PG8^T^ over time (Table 1). EV were observed for each production batch, with similar sizes ranging from 60 nm to 170 nm (with a few elements >170nm; Fig 1), and a density of around 30 EV/ 5 μm^2^ field (Table 1). Hence, our in-house experimental procedure confirmed the capacity of *A*. *laidlawii* PG8^T^ to produce EV and demonstrated for the first time and in a reproducible manner the production of EV by *Mmm* strain Afadé.

Because the diameter of the largest EV (from 170 to 220 nm in Fig 1) was very close to that of viable mycoplasma cells (from 300 to 1000 nm [15]) that could get smaller in nutritional stress conditions [25], the presence of viable cells in our EV preparations was checked by plating a quarter of each preparation on PPLO agar medium. No viable cell was detected in any production batch of *Mmm*, indicating that the largest EV are not small viable cells that could have been co-purified with EV, whereas 14 cells of *A*. *laidlawii* were found which corresponded to 56 cells in the whole batch (Table 1). Although this cell contamination was very limited (56 cells out of 1.8x10^10^ cfu in the whole culture after 96h incubation), it might affect the characterization of EV composition, notably regarding DNA/RNA content that relies on PCR amplifications. In consequence, we chose i) not to use a 0.1 μm filtering step that could potentially minimize cell contamination but might also retain some of the largest EV and ii) to systematically ascertain the quality of our EV preparation batches by performing a sterility control.

Microscopic observations of a *Mmm* Afadé culture in m-PPLO medium before EV purification showed a huge dispersion of spherical elements, including cells, and a clear overlapping area corresponding to EV (S1 Fig). Our EV isolation process resulted in a neat enrichment of the 60–170 nm EV and a depletion of the largest EV (170–220 nm, 3% in the EV preparation versus 13% in the *Mmm* culture; S1 Fig). Early microscopic observations showed membrane-surrounded vesicles (100–200 nm) named “elementary bodies” for several *Mycoplasma* species [26]. Such observations were confirmed by Robertson et al. on an aging culture of *M*. *hominis* in which small cell-like bodies (100–250 nm) associated in 0.22 μm-nonfilterable aggregates were evidenced [27]. Once dispersed by pronase treatment and transferred to favorable growth conditions these small cells turned out to be of spherical shape and viable, although poorly [27]. They were named at that time “elementary bodies”. Our EV isolation process involved a 0.22 μm filtration step and a viability control ensuring the depletion of such elementary bodies which suggests that the 3% largest structure in our EV preparation are really large EV and not elementary bodies resulting from aberrant cell divisions in unfavorable growth conditions.

### Universality of EV production by *Mycoplasma spp*.

**Fig 2:**
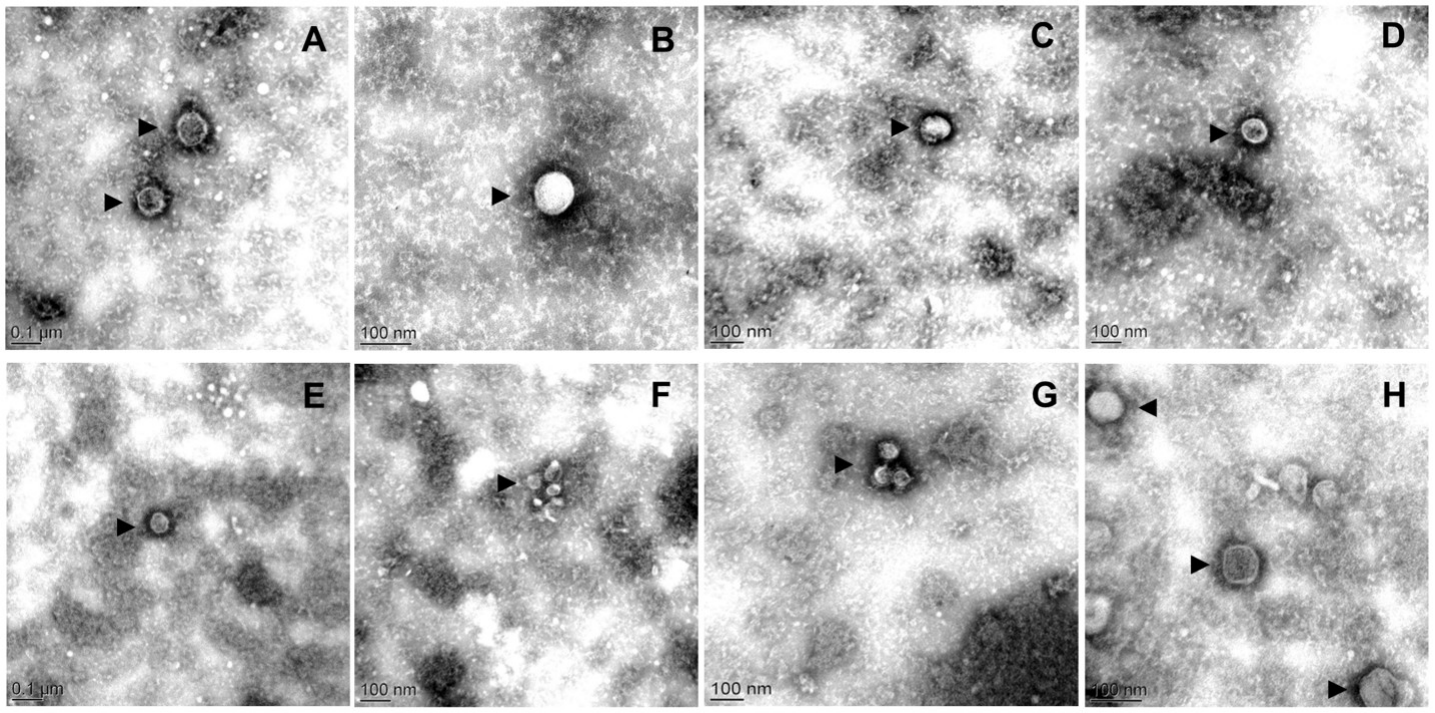
Electron micrographs of negatively-stained EV purified from *A*. *laidlaiwii* PG8^T^ (A), *M*. *mycoides* subsp. *mycoides* Afadé (B), *M*. *mycoides* subsp. *capri* PG3^T^ (C), *M*. *capricolum* subsp. *capricolum* L15937 (D), *M*. *agalactiae* 5632 (E), *M*. *agalactiae* L14628 (F), *M*. *bovis* L15762 (G) and *M*. *fermentans* PG18^T^ (H). Examples of EV are indicated by black arrowheads.

**Fig 3:**
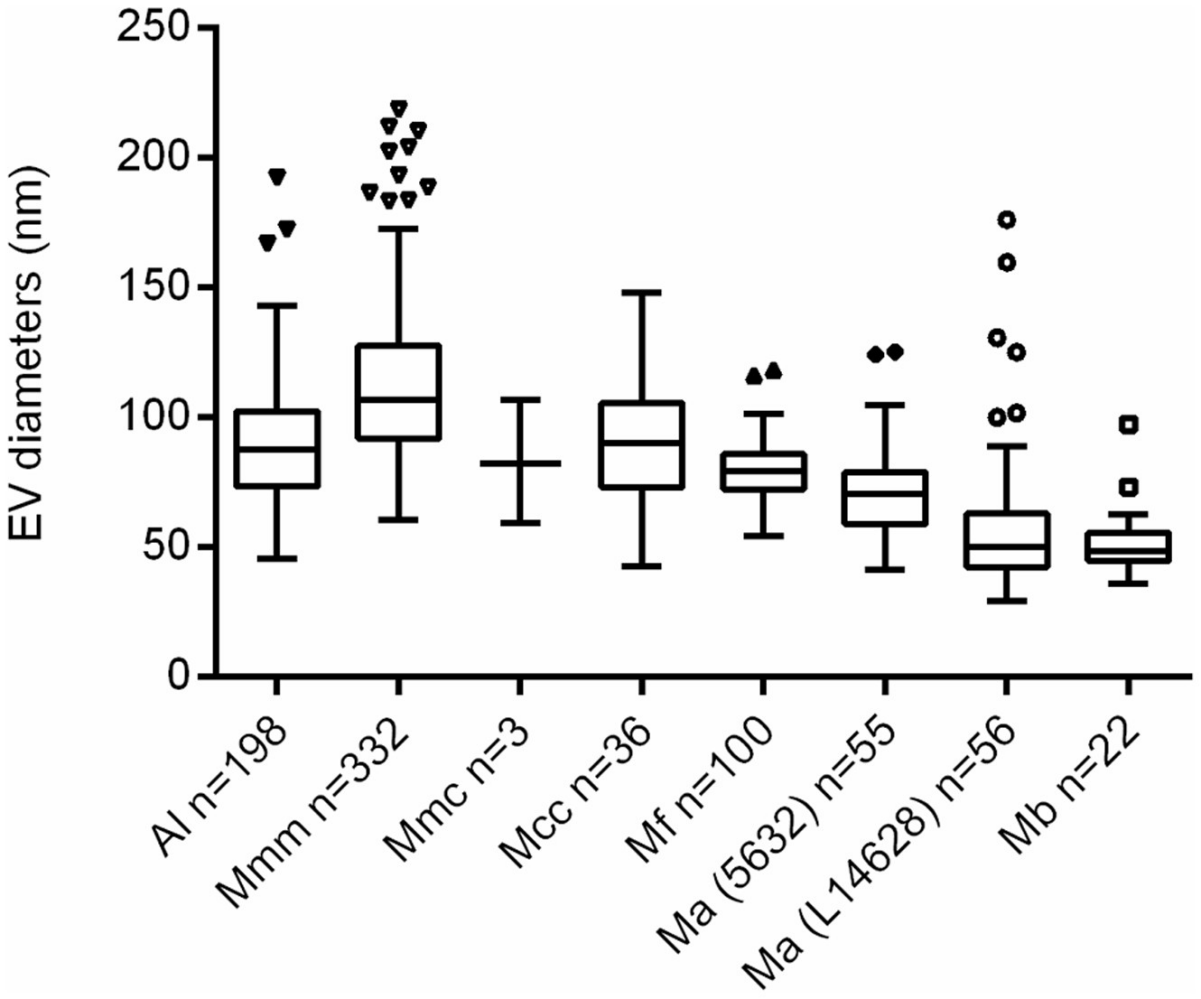
EV diameters from different (sub)species, as observed in electron micrographs (Tukey representation). Al, *A*. *laidlaiwii* PG8^T^; Mmm, *M*. *mycoides* subsp. *mycoides* Afadé; Mmc, *M*. *mycoides* subspp. *capri* PG3^T^; Mcc, *M*. *capricolum* subsp. *capricolum* L15937; Ma, *M*. *agalactiae*; Mb, *M*. *bovis* L15762; and Mf, *M*. *fermentans* PG18^T^. The number (n) of EV observed is indicated for each (sub)species. For Mmm, this “n” results from 3 batches of EV production.

Our validated protocol was used to test the capacity of 5 other *Mycoplasma* (sub)species, belonging to two different phylogenetic groups, to produce EV (Table 1). To take into consideration the different loss of titer between fast growing, acidifying species (*M*. *mycoides* subsp. *capri* and *M*. *capricolum* subsp. *capricolum)* and others, EV were isolated from all species from supernatants obtained after either 48h or 96h (see Table 1). Fig 2 shows the spherical shape of EV isolated from different mycoplasma (sub)species. The mean respective diameters varied both between species and within species, as illustrated for *M*. *agalactiae* (Fig 3). The smallest vesicles were observed for *M*. *agalactiae* strain L14628 and *M*. *bovis* strain L15762. *M*. *mycoides* subsp. *mycoides* Afadé produced the largest EV. Similarly, the EV production yield, estimated by counting the mean number of EV per microscopic field varied considerably between species, *M*. *fermentans* strain PG18^T^ and *M*. *mycoides* subsp. *mycoides* strain Afadé being the most productive under our experimental conditions (Table 1). Four out of seven EV preparations contained small amounts of viable cells (Table 1). This precluded any characterization of DNA/RNA content based on PCR techniques and further emphasized the difficulty of separating EV from minute sized cells characteristic of the *Mycoplasma* genus.

**Fig 4:**
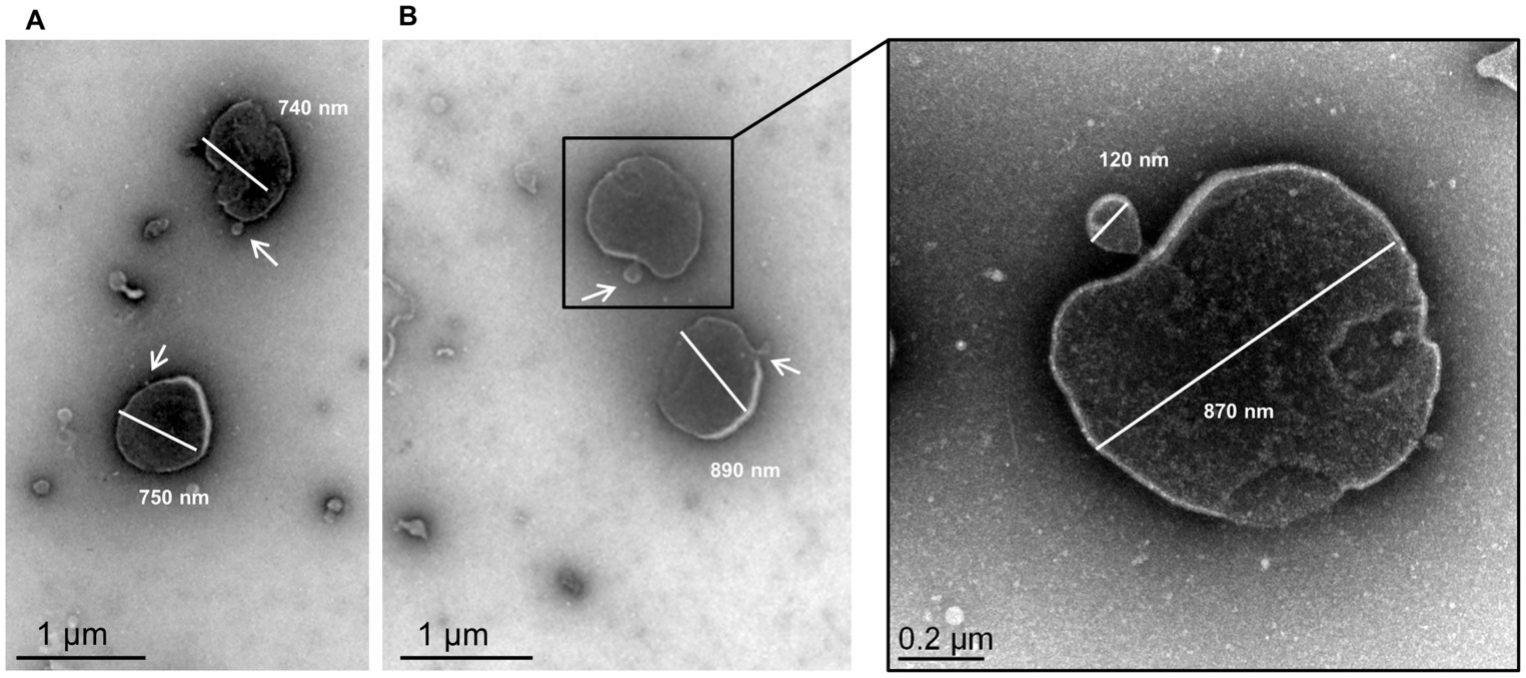
Transmission electron micrographs (A, B) of *M*. *mycoides* subsp. *mycoides* (strain Afadé) cells showing vesicle-like structures budding from the surface of the mycoplasma cells. A zoom of micrograph B is shown in the right panel. Diameters were estimated using imageJ.

Our m-PPLO medium inoculated with a high density of cells became suboptimal after 24h and viability started to decrease (loss of 1 to 3 log, Table 1). This raises the question of whether the observed EV might result from spontaneously reassembling membrane fragments of dead cells, *i*.*e*. could result from a degenerative process instead of being actively produced by viable cells. Two elements are in favor of an active EV production by viable cells. Firstly, the level of EV production is not related to cellular mortality in m-PPLO overtime but instead depends on species and strains: for instance, *M*. *fermentans* and *M*. *bovis* showed similar viable cell counts at 24h and 96h in m-PPLO although *M*. *fermentans* produced much more EV than *M*. *bovis*. Secondly, proteolipid vesicles artificially reconstituted from *M*. *fermentans* membranes were described as 1 μm-spheres [28], which is far larger than the EV observed here. This point was experimentally addressed by studying the EV production by heat- or gentamicin-killed *Mmm* strain Afadé cells. *Mmm* viability could be dramatically reduced by a 1h incubation at 60°C [29] or by treatment with 200 μg /ml gentamicin (i.e. 4-fold the MIC [30]). Indeed no viable cells were detected after heat treatment and a loss of 5 log was observed after 72h incubation with 200 μg/ml of gentamicin. EV production was quantified by immunodetection of the *Mmm* glucose permease (MSC_0860), a protein present in the membrane of *Mmm* EV (S1 Table) and specifically detected with high sensitivity by the 3f3 monoclonal antibody [19]. A correlation was indeed established between EV density estimated on electron micrographs and by the 3f3 detection by dot blotting (S2A Fig). Both heat and gentamicin treatment greatly reduced EV production (S2B Fig). This demonstrates that EV production relies on the presence of viable cells. Fig 4 further shows some putative vesicles budding from the cell surface of *Mmm* Afadé cells grown for 24h in m-PPLO medium. The size ratio between the cell and the vesicle is not characteristic of a binary fission and hence precludes the hypothesis of aberrant cell division.

Whatever the exact mechanism leading to EV release, it results in a multiplication of mycoplasma “forms/particles” that interact with the host cells. The protein content of EV-membranes was further analyzed to investigate its potential role in mycoplasma-host interactions.

### Identification of virulence factors among the EV membrane associated proteins

The three EV preparations from *Mycoplasma* (sub)species showing none or negligible viable cell contamination (Table 1), *i*.*e*. *M*. *mycoides* subsp. *mycoides* Afadé, *M*. *agalactiae* 5632 and *M*. *fermentans* PG18^T^, were chosen to perform proteomic analyses on Triton X-114 enriched fractions. In these fractions 88, 58 and 258 proteins were identified, respectively, of which 54%, 88% and 54% were membrane-bound or membrane proteins (S1 Table). However, in our hands, the protein concentration in each Triton extract was not equivalent for the 3 species and hence the relative abundance of each protein was hardly comparable between species (S1 Table). A semi-quantitative scale of the PAI was established to compare which proteins were most abundant in each preparation (PAI ≥1.0 = ++++; [0.5–1.0 [= +++; [0.1–0.5 [= ++ and a spectral count <0.1 = + (S1 Table)).

In order to demonstrate that the produced vesicles could play a role in the pathogenesis process, we highlighted all EV-membrane associated proteins which could be involved in the mycoplasma-host interactions (Table 2).

**Table 2:** List of EV membrane proteins potentially involved in mycoplasma-host interactions.

	Proteins	*M*. *mycoides* subsp. *mycoides* strain Afadé		*M*. *agalactiae* strain 5632		*M*. *fermentans* strain PG18^T^		Putative roles
		Uniprot n°	PAI	Uniprot n°	PAI	Uniprot n°	PAI	
**Lipoproteins**	Lipoprotein p37	**A0A0F2BJ67**	**+++**	**A5IY58**	**++**	**C4XFE4**	**++++**	Oncogenic
	Lipoprotein LppB and homologues	A0A126SRI9	++++	A5IZ93	+++	-	-	Major antigen
				A5IY11	++			
				A5IY23	++			
	Lipoprotein p48 / MALP-404	-	-	F5HGV8	++++	Q9RGX5	++++	Immune modulation
	Lipoprotein p80 and homologues	-	-	A5IYU2	++++	C4XER1	++++	Major antigen
	Oligopeptide ABC transporter oppA	Nd	Nd	A5IXN9	+++	C4XEL2	++++	Apoptotic
	Lipoprotein nuclease MAG5040	-	-	A5IYU3	++	C4XER2	++++	Major antigen, Host colonization
	Lipoprotein acid phosphatase	-	-	A5IYN5	+++	C4XF87	++	Host colonization
	Lipoprotein p29	-	-	-	-	Q49159	++++	Major antigen, adhesion
	Variable surface lipoprotein Y (VpmaY)	-	-	F5HDB1	+++	-	-	Major antigen, interaction with host extracellular matrix
	Variable surface lipoprotein A (vpmaX)	-	-	F5HIG3	++	-	-	Major antigen, interaction with host extracellular matrix
	Variable surface lipoprotein U (VpmaU)	-	-	F5HEE7	++	-	-	Major antigen, interaction with host extracellular matrix
	Predicted lipoprotein MAG1050	-	-	A5IXP4	++	-	-	Host colonization
	Lipoprotein p40	-	-	F5HEF4	++	-	-	Interaction with host extracellular matrix
	Lipoprotein LppA p72	A0A109WHL4	++	-	-	-	-	Major antigen, immune modulation
**Non-lipoproteins**	Elongation factor TU	**A0A0F2BJ16**	**++++**	**A5IYA9**	**++++**	**C4XEI5**	**++++**	Interaction with host extracellular matrix, immune modulation
	Chaperone protein DnaK (Hsp70)	**A0A0F2BNC8**	**++++**	**A5IXT5**	**+**	**C4XE63**	**++++**	Immune modulation
	Glyceraldehyde-3-phosphate dehydrogenase	A0A126SR57	+++	Nd	Nd	C4XF61	++++	Interaction with host extracellular matrix
	Pyruvate kinase	A0A0X8KSH0	+++	Nd	Nd	C4XEC6	++++	Interaction with host extracellular matrix
	Lactate dehydrogenase	A0A0F2BK13	+++	Nd	Nd	C4XEL0	+++	Interaction with host extracellular matrix
	Phosphoglycerate mutase	A0A126SQS4	++	Nd	Nd	C4XEM3	++++	Interaction with host extracellular matrix
	Transketolase	A0A0F2BGX7	+	Nd	Nd	C4XFT7	++	Interaction with host extracellular matrix
	PTS glucose permease ptsG	A0A0X8KVY6	++++	-	-	-	-	Major antigen, immune-modulation
	Pyruvate dehydrogenase E1 subunit α	A0A0F2BLA7	+++	-	-	Nd	Nd	Interaction with host extracellular matrix
	Pyruvate dehydrogenase E1 subunit β	A0A0F2BL90	++	-	-	Nd	Nd	Interaction with host extracellular matrix
	Hexose phosphate transport protein uhpT	Nd	Nd	A5IYT6	++	-	-	Host colonization

Uniprot n°: uniprot accession number. PAI: protein abundance index (++++: ≥1.0; +++: [0.5–1.0[; ++: [0.1–0.5[; +: <0.1). Nd, proteins not detected in the proteome; “-“,no homologous proteins were found in this (sub)species. Proteins retrieved in EV from the three species are in bold.

Lipoproteins, which are known to be potent stimulators of macrophage activity associated with pro-inflammatory cytokines release [31], represented a non-negligible fraction of EV-proteins, ranging from 10 to 38%. As EV are able to disseminate a long distance from the infection site [32], the presence of lipoproteins in EV could contribute to the inflammatory process associated with mycoplasmoses. We further attempted to identify the EV-associated lipoproteins within each species that could play a role in interaction with the host (immune modulation, host colonization, interaction with host extracellular matrix…) (Table 2). A few of these lipoproteins were present in EV membrane fractions of all 3 species, including the p37 lipoprotein, which was shown to be oncogenic in *M*. *fermentans* [33]. Its action might be augmented as a result of dissemination through EV. Similarly the OppA lipoprotein that can induce apoptosis [34] is found in abundant proportions in both *M*. *agalactiae* and *M*. *fermentans* EV. Several other lipoproteins were identified, that are known to be major antigens of the different species, such as LppB and homologues [35]. These lipoproteins also exhibit several immunomodulatory properties, *e*.*g*. the production of IFNγ by *Mmm* LppA [36]. Variable surface proteins of *M*. *agalactiae* (Vpmas), which are involved in high frequency phase variation and hence immune escape, were also recovered from EV [37]. These Vpmas have recently been characterized as major adhesins [38]. *M*. *agalactiae* EV also carry a lipoprotein nuclease MAG5040 which has been proposed to allow neutrophil extracellular trap escape [39]. Hence several mycoplasma lipoproteins found in EV have functions related to modulation of the host immune response and to host colonization.

A similar pattern was detected for other membrane-bound proteins that are not lipoproteins. This was the case of the Elongation factor Thermo unstable (Ef-Tu) which was one of the most abundant proteins in each EV preparation. Interestingly, Ef-Tu was also found to be one of the most abundant proteins in *Staphylococcus aureus* EV [6]. This moonlighting protein was shown to interact with components of the host extracellular matrix [40, 41]. Its presence in each of the 3 batches of EV could argue in favor of EV release being a non-stochastic process although it could also reflect the fact that Ef-Tu is one of the most abundant bacterial proteins [41]. Similarly, the chaperone Hsp70 (DnaK) was found in the 3 EV-proteomes and was one of the most abundant proteins in *Mmm* strain Afadé and *M*. *fermentans* PG18^T^ EV, based on spectral counts (Table 2 and S1 Table). Together with Ef-Tu, Hsp70 has been associated with antibody production, and cytokine secretion in *M*. *ovipneumoniae* [42]. Finally, *Mmm* Afadé and *M*. *fermentans* PG18^T^ EV contained several enzymes of general metabolism such as lactate dehydrogenase, pyruvate dehydrogenase E1 enzyme, glyceraldehyde dehydrogenase, pyruvate kinase, phosphoglycerate kinase and transketolase, also known to interact with components of the host extracellular matrix [40]. The EV produced by *Mmm* Afadé were also enriched in the glucose permease MSC_0860, which had a practical interest as it was used as a marker for EV semi-quantification (see here before).

Mycoplasma proteins transported by vesicular structures had already been observed by electron microscopy as early as 1964 [43]. The authors described globular elements measuring 10 to 100 nm and carrying the then called “not-cell bound antigens”. Our analysis of EV membrane proteins confirmed that EV could be such carrier of antigens and could correspond to the globular structures described by Eng and Froholm [43]. Further identification of the complete cargo (soluble proteins including other antigens and DNA/RNA) has yet to be achieved but will require further improvement of the EV purification process, with a 100% efficient elimination of cell contaminations and protein aggregates. The use of gradient-based purification method might be a promising way [44].

## Conclusions

Fifty years ago microscopic observations led to the conclusion that mycoplasma replicating cells had a minimal diameter of 300 nm. Vesicular structures such as “globular elements” (10–100 nm) and “elementary bodies” (100–250 nm) were also observed, the latter being considered the results of aberrant cell division or reassembling of membrane fragments. The concept of extracellular vesicles actively secreted by bacteria was just starting to emerge at that time and mainly for Gram negative. Here, we showed that EV can be released by several *Mycoplasma* species, from different phylogenetic clades, and are produced by living cells in a budding-way compatible with the canonical definition of bacterial EV. The sizes of mycoplasma EV vary considerably (from 30 to 220 nm) and setting a size cut-off between small viable cells and vesicles is difficult. Nonetheless the vesicles purified in this study are different from the elementary bodies described by Robertson as the latter are removed by 0.22 μm filtration [27]. The recurring presence of homologous proteins in EV-membranes preparations from 3 *Mycoplasma* species, further contributes to characterize EV release as an active process that could constitute a new “secretion” pathway and hence account for some of the proteins recovered in mycoplasma secretomes. For instance, the secretome from *M*. *hyopneumoniae* has several proteins in common with the EV-proteome described here [45]. Although it has to be formally demonstrated in the host context, the release of EV could considerably multiply mycoplasma interaction with the host cells. Mycoplasma EV as a potential component of the interactome will require further attention for a better understanding of mycoplasma pathogenicity and also for vaccine development.

## Supporting information

S1 Fig Comparison of size distribution of EV (n = 322, three production batches) purified from *M*. *mycoides* subsp. *mycoides* Afadé (black bars) and cells (n = 124) from a stationary phase culture of *M*. *mycoides* subsp. *mycoides* Afadé (white bars). EV and cells were observed by TEM after negative staining and diameter was estimated using ImageJ.
(TIF)

S2 Fig **A. Validation of 3f3 dot blotting for EV semi-quantification.** EV were extracted from 6h, 48h and 96h cultures of *M*. *mycoides* subsp. *mycoides* strain Afadé in m-PPLO medium. Three production batches were done for each time point. EV are indicated by black arrowheads on electron micrographs. Their density was estimated by counting the number of EV per 5 μm^2^ fields (scale: +/-, counts <1 EV/ 5μm^2^; +, 1–10 EV/ 5μm^2^; ++, 10–100 EV/ 5μm^2^). Dot blotting with 3f3 was performed on 2μl EV extract for each time point and each batch. The intensity of dot blotting correlates with the EV density in electron micrographs. **B. Requirement of *Mycoplasma* viable cells for EV production.** *M*. *mycoides* subsp. *mycoides* strain Afadé cultivated in m-PPLO medium (10^8^–10^9^ cfu/ml) was submitted to heat (1h at 60°C, no more viable cells) or chemical (200 μg/ml gentamicin, viability was reduced to 10^2^ and 10^7^ cfu/ml with or without gentamicin, respectively) inactivation before EV purification (for details see the Materials and methods section). EV (indicated by black arrowheads) density was estimated by counting on electron micrographs and/or by 3f3 dot-blotting (see panel A). When mycoplasma cells were killed no EV were produced.
(TIF)

S1 Table Proteins identified from the Triton X-114 fractions of EV. Proteins are classified according to their PAI.
(DOCX)
