# Supplementary figures and images for: Mycoplasmas are no exception to extracellular vesicles release: Revisiting old concepts
Source: PLoS One. 2018 Nov 28;13(11):e0208160. doi: 10.1371/journal.pone.0208160 (PMC6261642; doi:10.1371/journal.pone.0208160)

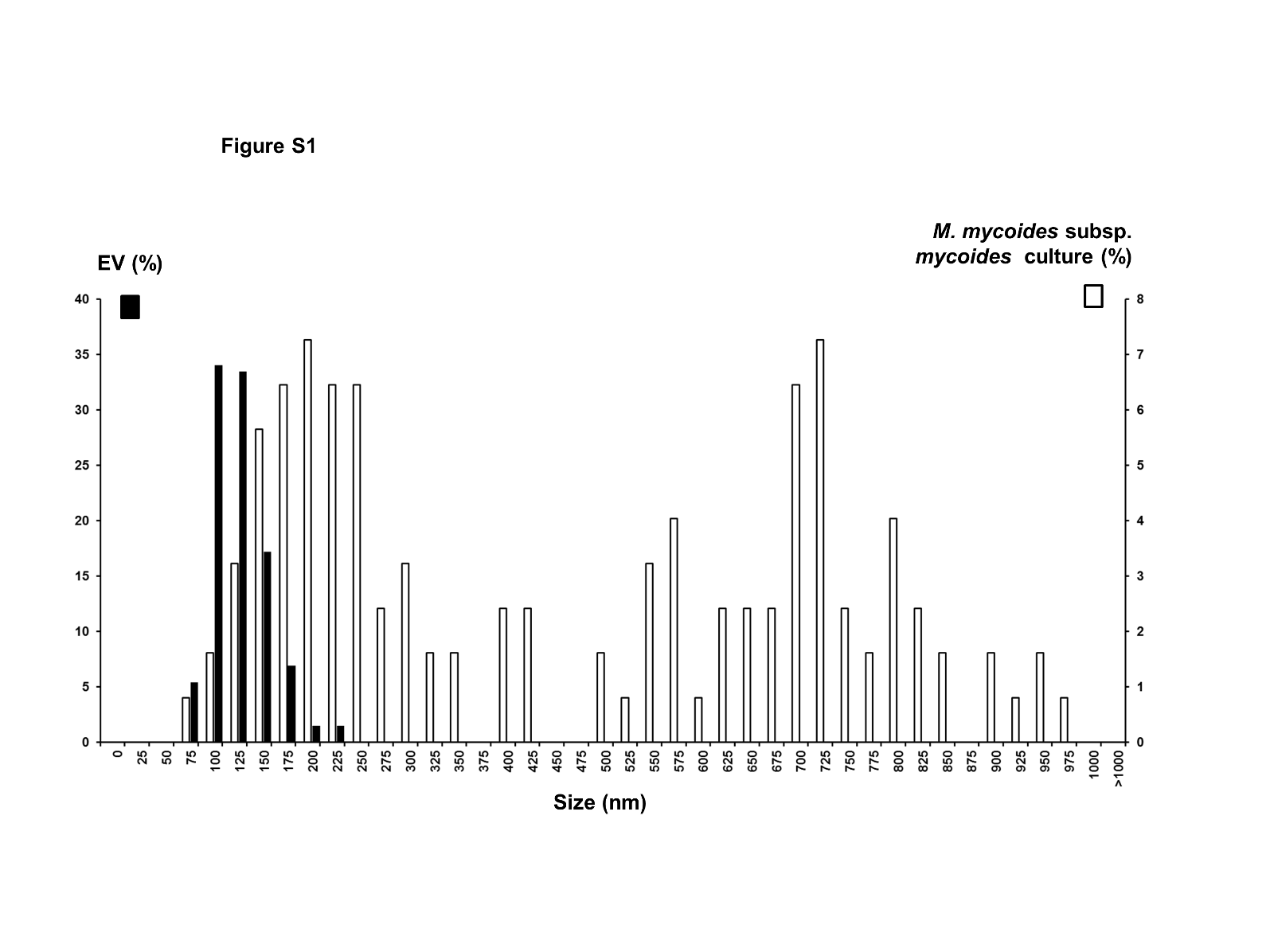

Supplement: S1 Fig — EV and cells were observed by TEM after negative staining and diameter was estimated using ImageJ. (TIF) [file pone.0208160.s001.tif]

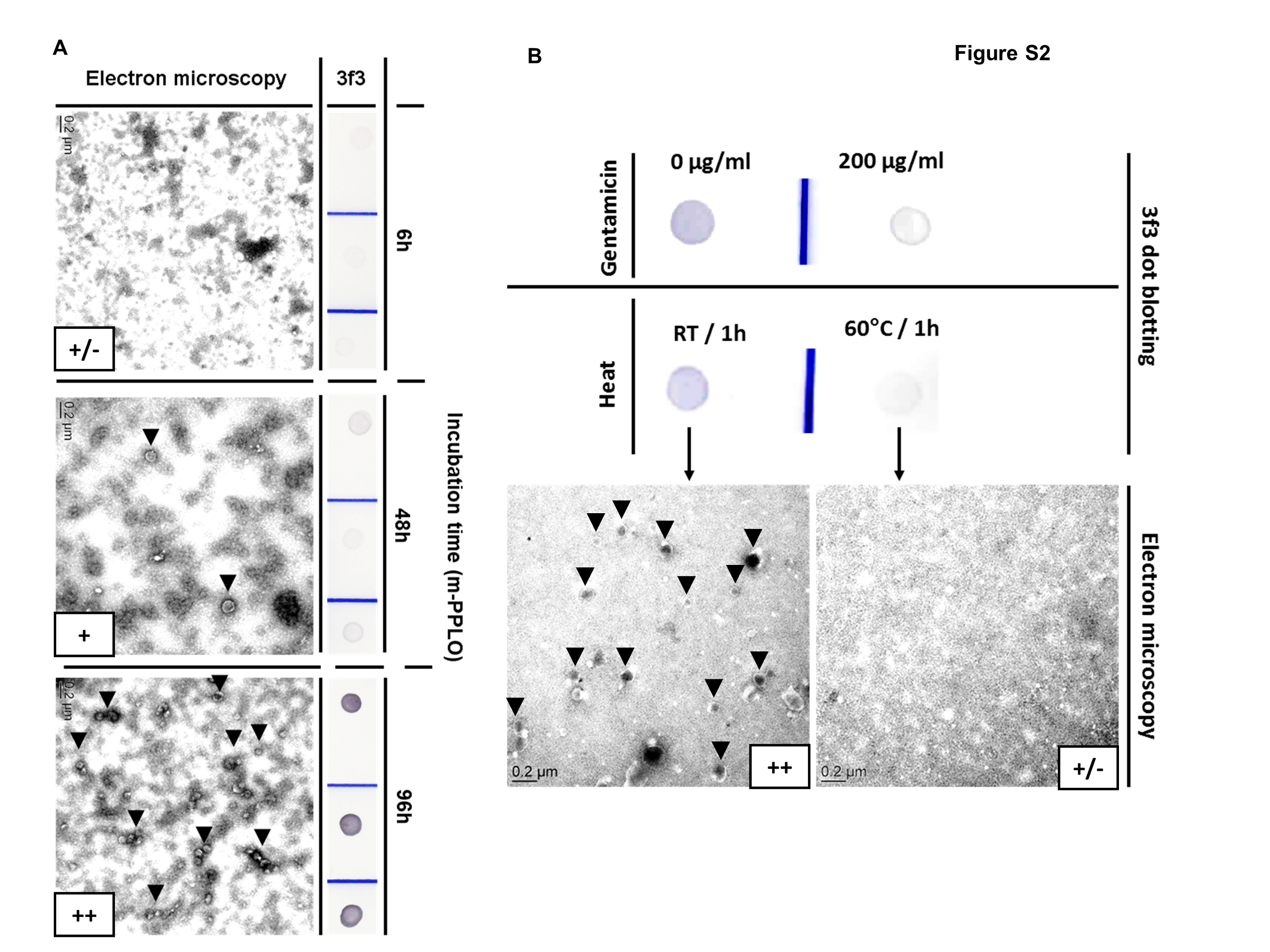

Supplement: S2 Fig — A. Validation of 3f3 dot blotting for EV semi-quantification. EV were extracted from 6h, 48h and 96h cultures of M. mycoides subsp. mycoides strain Afadé in m-PPLO medium. Three production batches were done for each time point. EV are indicated by black arrowheads on electron micrographs. Their density was estimated by counting the number of EV per 5 μm2 fields (scale: +/-, counts <1 EV/ 5μm2; +, 1–10 EV/ 5μm2; ++, 10–100 EV/ 5μm2). Dot blotting with 3f3 was performed on 2μl EV extract for each time point and each batch. The intensity of dot blotting correlates with the EV density in electron micrographs. B. Requirement of Mycoplasma viable cells for EV production. M. mycoides subsp. mycoides strain Afadé cultivated in m-PPLO medium (108–109 cfu/ml) was submitted to heat (1h at 60°C, no more viable cells) or chemical (200 μg/ml gentamicin, viability was reduced to 102 and 107 cfu/ml with or without gentamicin, respectively) inactivation before EV purification (for details see the Materials and methods section). EV (indicated by black arrowheads) density was estimated by counting on electron micrographs and/or by 3f3 dot-blotting (see panel A). When mycoplasma cells were killed no EV were produced. (TIF) [file pone.0208160.s002.tif]
