# Supplementary material for: Mycoplasmas are no exception to extracellular vesicles release: Revisiting old concepts
Source: PLoS One. 2018 Nov 28;13(11):e0208160. doi: 10.1371/journal.pone.0208160 (PMC6261642; doi:10.1371/journal.pone.0208160)

**Table S1.** Proteins identified from the Triton X-114 fractions of EV. Proteins are classified according to their PAI.

***M. agalactiae* 5632 (Triton X-114 fraction)**

| **Uniprot accesion number** | **Molligen accesion number** | **predicted funtion** | **log(E value)** | **Coverage** | **MW** | **size** | **Spectra** | **Specific** | **Uniques** | **Specific uniques** | **Uniques peptide-mod-charge** | **Theoretical number of tryptic peptides** | **PAI** |  |
| --- | --- | --- | --- | --- | --- | --- | --- | --- | --- | --- | --- | --- | --- | --- |
| F5HGV8 | MAG0120 | predicted lipoprotein P48 | -182,42 | 40 | 51 | 465 | 53 |  | 24 |  | 31 | 22 | 2,41 | ++++ |
| A5IZ32 | MAG5910 | bifunctional metallophosphatase/5'-nucleotidase (1 TMD) | -183,94 | 45 | 76 | 681 | 47 |  | 36 |  | 42 | 26 | 1,81 | ++++ |
| A5IYA9 | MAG3200 | **elongation factor TU** | -90,47 | 30 | 43 | 396 | 25 |  | 14 |  | 15 | 20 | 1,25 | ++++ |
| A5IYU2 | MAG5030 | lipoprotein P80 | -147,48 | 30 | 81 | 721 | 41 |  | 24 |  | 29 | 37 | 1,11 | ++++ |
| A5IXY4 | MAG1950 | 30S ribosomal protein S20 rpsT | -4,64 | 23 | 10 | 89 | 2 |  | 2 |  | 2 | 2 | 1,00 | ++++ |
| A5IYE9 | MAG3600 | hypothetical membrane protein (1 TMD) | -41,19 | 36 | 22 | 194 | 10 |  | 9 |  | 9 | 11 | 0,91 | +++ |
| A5IYN5 | MAG4460 | lipoprotein acid phosphatase | -55,13 | 17 | 52 | 462 | 14 |  | 11 |  | 12 | 16 | 0,88 | +++ |
| A5IYR3 | MAG4740 | Hypothetical protein, predicted lipoprotein | -27,05 | 22 | 25 | 222 | 7 |  | 6 |  | 6 | 8 | 0,88 | +++ |
| A5IYE7 | MAG3580 | hypothetical membrane protein (7 TMD) | -21,66 | 14 | 35 | 304 | 5 |  | 4 |  | 4 | 6 | 0,83 | +++ |
| A5IXR1 | MAG1220 | hypothetical membrane protein (1 TMD), lemA family protein | -56,76 | 38 | 26 | 232 | 12 |  | 11 |  | 12 | 15 | 0,80 | +++ |
| A5IXQ7 | MAG1180 | XAA-PRO aminopeptidase | -29,50 | 16 | 40 | 350 | 8 |  | 5 |  | 6 | 10 | 0,80 | +++ |
| A5IXN9 | MAG1000 | oligopeptide ABC transporter oppA (lipoprotein) | -151,54 | 27 | 110 | 959 | 32 |  | 27 |  | 29 | 42 | 0,76 | +++ |
| A5IXN8 | MAG0990 | hypothetical membrane protein (5 TMD) | -22,36 | 14 | 35 | 299 | 7 |  | 5 |  | 6 | 10 | 0,70 | +++ |
| A5IZ93 | MAG6520 | Lipoprotein LppB homolog | -109,67 | 26 | 70 | 621 | 21 |  | 16 |  | 19 | 32 | 0,66 | +++ |
| F5HDB1 | MAG7080 | Variable surface lipoprotein Y (VpmaY) | -41,43 | 17 | 37 | 346 | 9 | 8 | 7 | 6 | 7 | 14 | 0,64 | +++ |
| A5IXJ8 | MAG0590 | hypothetical membrane protein (5 TMD) OxaA/YidC family | -114,19 | 31 | 83 | 715 | 21 |  | 20 |  | 20 | 33 | 0,64 | +++ |
| A5IZ61 | MAG6200 | predicted lipoprotein (leucine-rich repeat protein) | -16,09 | 12 | 27 | 241 | 6 |  | 5 |  | 6 | 10 | 0,60 | +++ |
| A5IXF3 | MAG0150 | Sugar ABC transporter Permease | -42,63 | 12 | 74 | 662 | 12 |  | 11 |  | 11 | 21 | 0,57 | +++ |
| A5IY98 | MAG3090 | hypothetical membrane protein (1 TMD) | -37,31 | 20 | 36 | 314 | 8 |  | 7 |  | 8 | 15 | 0,53 | +++ |
| A5IYU3 | MAG5040 | lipoprotein nuclease MAG5040 | -39,18 | 21 | 45 | 390 | 9 |  | 8 |  | 9 | 19 | 0,47 | ++ |
| A5IY11 | MAG2220 | lipoprotein LppB homolog | -34,96 | 17 | 69 | 617 | 11 | 10 | 10 | 9 | 11 | 24 | 0,46 | ++ |
| F5HIG3 | MAG7070 | Variable surface lipoprotein A (vpmaX) | -13,74 | 13 | 25 | 221 | 4 | 3 | 3 | 2 | 4 | 9 | 0,44 | ++ |
| A5IXE9 | MAG0110 | hypothetical membrane protein (7 TMD) | -12,30 | 14 | 31 | 273 | 3 |  | 3 |  | 3 | 7 | 0,43 | ++ |
| A5IYV6 | MAG5170 | Energy-coupling factor transporter ATP-binding protein EcfA2 | -17,62 | 7 | 35 | 311 | 5 |  | 5 |  | 5 | 12 | 0,42 | ++ |
| A5IYT6 | MAG4970 | hexosephosphate transport protein uhpT (12 TMD) | -13,04 | 4 | 54 | 498 | 3 |  | 3 |  | 3 | 8 | 0,38 | ++ |
| A5IZE4 | MAG7030 | ECF transporter S component (7 TMD, DUF1393) | -8,39 | 8 | 38 | 335 | 4 |  | 3 |  | 3 | 11 | 0,36 | ++ |
| A5IY24 | MAG2350 | predicted lipoprotein | -17,86 | 10 | 40 | 356 | 4 |  | 3 |  | 4 | 12 | 0,33 | ++ |
| A5IXX0 | MAG1810 | hypothetical membrane protein (4 TMD) | -30,07 | 14 | 53 | 472 | 6 |  | 5 |  | 5 | 18 | 0,33 | ++ |
| A5IXG6 | MAG0280 | hypothetical membrane protein (6 TMD) | -27,42 | 9 | 75 | 655 | 8 |  | 7 |  | 8 | 26 | 0,31 | ++ |
| A5IZG3 | MAG7220 | **1-acyl-SN-glycerol-3-phosphate acyltransferase PlsC** | -16,02 | 15 | 28 | 246 | 4 |  | 4 |  | 4 | 13 | 0,31 | ++ |
| A5IY57 | MAG2680 | hypothetical membrane protein (1 TMD) | -20,12 | 14 | 56 | 490 | 6 |  | 6 |  | 6 | 20 | 0,30 | ++ |
| A5IY70 | MAG2810 | hypothetical membrane protein (2 TMD) | -15,06 | 9 | 48 | 416 | 5 |  | 5 |  | 5 | 17 | 0,29 | ++ |
| A5IY63 | MAG2740 | Alcohol dehydrogenase | -21,18 | 12 | 37 | 348 | 4 |  | 4 |  | 4 | 14 | 0,29 | ++ |
| F5HEE7 | MAG7090 | Variable surface lipoprotein U (VpmaU) | -12,37 | 5 | 25 | 238 | 3 |  | 2 |  | 3 | 11 | 0,27 | ++ |
| A5IY50 | MAG2610 | lipoprotein, peptidase family S41 | -44,03 | 10 | 71 | 625 | 7 |  | 6 |  | 7 | 26 | 0,27 | ++ |
| A5IY23 | MAG2340 | lipoprotein LppB homolog | -34,54 | 14 | 66 | 586 | 7 | 6 | 7 | 6 | 7 | 27 | 0,26 | ++ |
| A5IXP4 | MAG1050 | predicted lipoprotein | -17,66 | 13 | 37 | 328 | 4 |  | 4 |  | 4 | 16 | 0,25 | ++ |
| A5IZ59 | MAG6180 | 50S ribosomal protein L7/L12 rplL | -6,84 | 18 | 13 | 123 | 2 |  | 2 |  | 2 | 8 | 0,25 | ++ |
| A5IYU7 | MAG5080 | predicted lipoprotein | -30,68 | 14 | 59 | 510 | 6 |  | 6 |  | 6 | 25 | 0,24 | ++ |
| A5IYG9 | MAG3800 | hypothetical membrane protein (1 TMD) | -5,45 | 8 | 26 | 228 | 3 |  | 3 |  | 3 | 13 | 0,23 | ++ |
| A5IXT4 | MAG1450 | predicted lipoprotein | -5,43 | 8 | 35 | 317 | 4 |  | 3 |  | 4 | 18 | 0,22 | ++ |
| A5IXR0 | MAG1210 | hypothetical membrane protein (2 TMD) | -10,66 | 6 | 70 | 606 | 5 |  | 4 |  | 5 | 23 | 0,22 | ++ |
| A5IXW7 | MAG1780 | hypothetical membrane protein (2 TMD) | -7,44 | 6 | 38 | 331 | 3 |  | 2 |  | 2 | 14 | 0,21 | ++ |
| A5IXT2 | MAG1430 | hypothetical membrane protein (1 TMD) | -16,60 | 8 | 56 | 495 | 4 |  | 4 |  | 4 | 19 | 0,21 | ++ |
| A5IY81 | MAG2920 | hypothetical membrane protein (2 TMD) | -30,31 | 9 | 83 | 748 | 6 |  | 6 |  | 6 | 29 | 0,21 | ++ |
| A5IYY3 | MAG5430 | **50S ribosomal protein L2 rplB** | -4,14 | 6 | 31 | 281 | 2 |  | 2 |  | 2 | 10 | 0,20 | ++ |
| A5IY58 | MAG2690 | **lipoprotein p37** | -17,95 | 7 | 50 | 438 | 4 |  | 4 |  | 4 | 21 | 0,19 | ++ |
| A5IYP2 | MAG4530 | hypothetical membrane protein (2 TMD) | -13,62 | 7 | 53 | 442 | 4 |  | 3 |  | 3 | 21 | 0,19 | ++ |
| A5IYV7 | MAG5180 | ABC transporter ATP-binding protein | -8,04 | 7 | 29 | 265 | 2 |  | 2 |  | 2 | 12 | 0,17 | ++ |
| F5HEF4 | MAG2410 | lipoprotein P40 | -10,39 | 5 | 40 | 359 | 3 |  | 2 |  | 2 | 18 | 0,17 | ++ |
| A5IYU6 | MAG5070 | ABC transporter permease protein (6 TMD) | -4,18 | 5 | 38 | 327 | 2 |  | 2 |  | 2 | 13 | 0,15 | ++ |
| A5IZG9 | MAG7280 | Chaperone protein DnaJ | -3,24 | 9 | 41 | 376 | 2 |  | 2 |  | 2 | 14 | 0,14 | ++ |
| A5IYV4 | MAG5150 | predicted lipoprotein MAG1050 | -5,79 | 5 | 38 | 332 | 2 |  | 2 |  | 2 | 15 | 0,13 | ++ |
| A5IYV5 | MAG5160 | ABC/ECF transporter, transmembrane component (5 TMD) | -8,33 | 6 | 35 | 301 | 2 |  | 2 |  | 2 | 16 | 0,13 | ++ |
| A5IYV9 | MAG5200 | **DNA-directed RNA polymerase alpha chain** | -4,45 | 8 | 37 | 336 | 2 |  | 2 |  | 2 | 19 | 0,11 | ++ |
| A5IXT5 | MAG1460 | **Chaperone protein DnaK HSP70** | -7,08 | 5 | 65 | 598 | 2 |  | 2 |  | 2 | 22 | 0,09 | + |
| A5IXY7 | MAG1980 | predicted lipoprotein P60 | -7,11 | 5 | 54 | 461 | 2 |  | 2 |  | 2 | 26 | 0,08 | + |
| A5IYR7 | MAG4780 | putative ATP binding protein | -19,58 | 2 | 124 | 1071 | 3 |  | 3 |  | 3 | 53 | 0,06 | + |

| lipoproteins and transmembrane proteins |
| --- |
| membrane associated proteins |
| cytoplasmic proteins |
| PAI semi quantitative scale: ++++: ≥1.0; +++: [0.5-1.0[; ++: [0.1-0.5[; + <0.1. |
| Proteins retrieved in EV from the three species are in bold |

|  | **membrane** | **membrane-bound** | **cytoplasmic** |
| --- | --- | --- | --- |
| ***M. agalactiae* 5632 EV** | **46,00** | **5** | **7** |
| **58 proteins** |  |  |  |
| **percentage** | **79,31** | **9** | **12** |


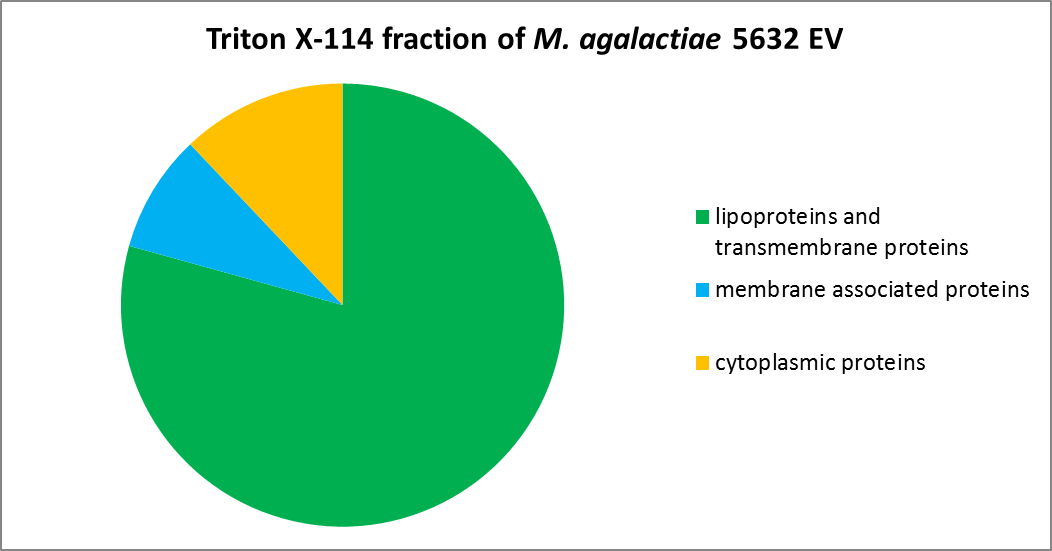


***M. mycoides* subsp. *mycoides* Afadé (Triton X-114 fraction)**

| **Uniprot accession number** | **molligen accession number** | **Predicted function** | **log (E value)** | **Coverage** | **MW** | **size** | **Spectra** | **Specific** | **Uniques** | **Specific uniques** | **Uniques peptide-mod-charge** | **Theoretical number of tryptic peptides** | **PAI** |  |
| --- | --- | --- | --- | --- | --- | --- | --- | --- | --- | --- | --- | --- | --- | --- |
| A0A126SRI9 | MSC_0519 | lipoprotein LppB | -717,20 | 76 | 70 | 622 | 158 |  | 106 |  | 135 | 30 | 5,27 | ++++ |
| A0A0F2BPZ9 | MSC_0079 | ABC transporter phosphonate (lipoprotein) | -357,42 | 63 | 50 | 447 | 100 |  | 53 |  | 67 | 24 | 4,17 | ++++ |
| A0A0F2BJ77 | MSC_0957 | predicted lipoprotein | -244,18 | 47 | 49 | 433 | 59 |  | 39 |  | 49 | 22 | 2,68 | ++++ |
| A0A126SRQ1 | MSC_0397 | predicted lipoprotein | -46,87 | 37 | 26 | 237 | 13 |  | 9 |  | 12 | 6 | 2,17 | ++++ |
| A0A0X8KVY6 | MSC_0860 | glucose permease PTS ptsG | -73,70 | 20 | 73 | 676 | 24 |  | 19 |  | 20 | 18 | 1,33 | ++++ |
| A0A0F2BJ16 | MSC_0160 | **Elongation factor TU** | -81,83 | 42 | 43 | 395 | 20 |  | 17 |  | 19 | 20 | 1,00 | ++++ |
| A0A0F2BNC8 | MSC_0610 | **chaperone protein DnaK Hsp70** | -109,86 | 27 | 64 | 591 | 21 |  | 17 |  | 18 | 21 | 1,00 | ++++ |
| A0A0F2BLJ1 | MSC_0257 | glycerol uptake facilitator factor permease | -19,09 | 14 | 27 | 259 | 5 |  | 4 |  | 4 | 5 | 1,00 | ++++ |
| A0A0F2BLA7 | MSC_0265 | Pyruvate dehydrogenase E1 component subunit alpha | -38,73 | 27 | 42 | 370 | 14 |  | 11 |  | 13 | 16 | 0,88 | +++ |
| A0A126SR57 | MSC_0679 | Glyceraldehyde-3-phosphate dehydrogenase | -66,62 | 28 | 37 | 337 | 14 |  | 12 |  | 13 | 17 | 0,82 | +++ |
| A0A0F2BPV7 | MSC_0588 | Cell division protein FtsZ | -76,85 | 24 | 41 | 386 | 11 |  | 10 |  | 11 | 14 | 0,79 | +++ |
| A0A0F2BK13 | MSC_0532 | L-lactate dehydrogenase | -44,13 | 28 | 35 | 318 | 10 |  | 10 |  | 10 | 13 | 0,77 | +++ |
| A0A0F2BN33 | MSC_0678 | Phosphoglycerate kinase | -65,07 | 34 | 44 | 404 | 15 |  | 14 |  | 15 | 20 | 0,75 | +++ |
| A0A0F2BI99 | MSC_0112 | Acyl-phosphate:glycerol-3-phosphate O-acyltransferase PlsY (7 TMD) | -9,63 | 9 | 29 | 253 | 2 |  | 2 |  | 2 | 3 | 0,67 | +++ |
| A0A0F2BJ67 | MSC_0790 | **Lipoprotein p37** | -68,08 | 24 | 56 | 490 | 13 |  | 13 |  | 13 | 20 | 0,65 | +++ |
| A0A126SR61 | MSC_0575 | predicted lipoprotein | -63,56 | 37 | 39 | 339 | 12 |  | 12 |  | 12 | 19 | 0,63 | +++ |
| A0A126SRA1 | MSC_0671 | hypothetical membrane protein (7 TMD) | -21,31 | 10 | 36 | 314 | 5 |  | 5 |  | 5 | 8 | 0,63 | +++ |
| A0A120KUK9 | MSC_0163 | leucine aminopeptidase | -54,92 | 20 | 50 | 451 | 12 |  | 9 |  | 10 | 21 | 0,57 | +++ |
| A0A0F2BHF2 | MSC_0607b/608 | 30S ribosomel protein S2 | -16,95 | 15 | 33 | 292 | 5 |  | 5 |  | 5 | 9 | 0,56 | +++ |
| A0A126SQQ3 | MSC_0728 | 30S ribosomal protein S5 | -27,65 | 22 | 28 | 254 | 6 |  | 5 |  | 6 | 11 | 0,55 | +++ |
| A0A0F2BP06 | MSC_0742 | **50S ribosomal protein L2** | -11,41 | 14 | 31 | 282 | 4 |  | 4 |  | 4 | 8 | 0,50 | +++ |
| A0A0F2BI79 | MSC_0278 | 30S ribosomal protein S4 | -11,96 | 16 | 24 | 208 | 5 |  | 4 |  | 4 | 10 | 0,50 | +++ |
| A0A126SUD1 | MSC_0334 | histidyl-tRNA synthetase | -29,85 | 19 | 48 | 414 | 9 |  | 8 |  | 9 | 20 | 0,45 | ++ |
| A0A0F2BHR4 | MSC_0011 | ribose/galactose ABC transporter | -51,70 | 17 | 61 | 550 | 11 |  | 11 |  | 11 | 25 | 0,44 | ++ |
| A0A126SSH7 | MSC_0258 | glycerol kinase | -62,94 | 19 | 57 | 505 | 11 |  | 9 |  | 10 | 27 | 0,41 | ++ |
| A0A0F2BGY7 | MSC_0739 | 30S ribosomal protein S3 | -17,90 | 14 | 26 | 233 | 4 |  | 4 |  | 4 | 10 | 0,40 | ++ |
| A0A0F2BLE2 | MSC_0263 | NADH oxydase | -19,16 | 12 | 50 | 454 | 8 |  | 6 |  | 7 | 20 | 0,40 | ++ |
| A0A0F2BNK0 | MSC_0453 | trigger factor (prolyl isomerase) | -47,37 | 22 | 49 | 428 | 10 |  | 9 |  | 10 | 26 | 0,38 | ++ |
| A0A126SQY0 | MSC_0757 | transmembrane protein (3 TMD) tetraspanin family | -17,11 | 11 | 29 | 253 | 3 |  | 3 |  | 3 | 8 | 0,38 | ++ |
| A0A0F2BL90 | MSC_0266 | Pyruvate dehydrogenase E1 component subunit beta | -12,60 | 12 | 36 | 329 | 5 |  | 4 |  | 5 | 14 | 0,36 | ++ |
| A0A0F2BNX6 | MSC_0830 | thymidine phosphorylase | -32,10 | 19 | 49 | 437 | 8 |  | 7 |  | 8 | 23 | 0,35 | ++ |
| A0A0F2BQ61 | MSC_0029 | FMN-dependent NADH-azoreductase | -19,18 | 15 | 22 | 199 | 3 |  | 3 |  | 3 | 9 | 0,33 | ++ |
| A0A0F2BPN1 | MSC_0256 | Hypoxanthine phosphoribosyltransferase | -3,71 | 10 | 22 | 190 | 2 |  | 2 |  | 2 | 6 | 0,33 | ++ |
| A0A0X8KSH0 | MSC_0261 | pyruvate kinase | -21,79 | 12 | 54 | 478 | 6 |  | 6 |  | 6 | 19 | 0,32 | ++ |
| A0A126SSP3 | MSC_0134 | CTP synthase | -28,70 | 14 | 60 | 532 | 8 |  | 7 |  | 8 | 26 | 0,31 | ++ |
| A0A0F2BG74 | MSC_0995 | 50S ribosomal proteine L1 | -10,84 | 15 | 25 | 226 | 3 |  | 3 |  | 3 | 10 | 0,30 | ++ |
| A0A0F2BIG2 | MSC_0030 | ABC transporter ATP binding protein | -12,58 | 12 | 39 | 344 | 5 |  | 5 |  | 5 | 17 | 0,29 | ++ |
| A0A0F2BJU1 | MSC_0744 | 50S ribosomal protein L4 | -10,16 | 7 | 23 | 208 | 2 |  | 2 |  | 2 | 7 | 0,29 | ++ |
| A0A126SS55 | MSC_0349 | infB IF2 | -38,50 | 13 | 69 | 620 | 8 |  | 8 |  | 8 | 28 | 0,29 | ++ |
| A0A0F2BKN6 | MSC_0005 | hypothetical membrane protein (3 TMD) | -10,68 | 6 | 43 | 363 | 4 |  | 3 |  | 4 | 15 | 0,27 | ++ |
| A0A0F2BJI4 | MSC_0527 | lipoate protein ligase A | -9,77 | 9 | 40 | 345 | 4 |  | 3 |  | 3 | 16 | 0,25 | ++ |
| A0A0F2BIN6 | MSC_0505 | glycose 6 P isomerase | -29,13 | 10 | 48 | 427 | 4 |  | 4 |  | 4 | 17 | 0,24 | ++ |
| A0A0F2BL88 | MSC_0689 | hypothetical protein degV family | -7,19 | 9 | 32 | 279 | 3 |  | 3 |  | 3 | 13 | 0,23 | ++ |
| A0A140DZP8 | MSC_0971/978 | UDP glucose 4 epimerase | -19,43 | 13 | 29 | 251 | 3 |  | 3 |  | 3 | 13 | 0,23 | ++ |
| A0A0F2BMQ2 | MSC_0276 | dihydroxyacetone kinase | -3,26 | 11 | 23 | 208 | 2 |  | 2 |  | 2 | 9 | 0,22 | ++ |
| A0A0F2BMM0 | MSC_0732 | 50S ribosomal protein L5 | -15,42 | 12 | 20 | 180 | 2 |  | 2 |  | 2 | 9 | 0,22 | ++ |
| A0A0F2BMD6 | MSC_0080 | arginine tRNA syntethase | -18,48 | 10 | 52 | 454 | 5 |  | 5 |  | 5 | 23 | 0,22 | ++ |
| A0A0F2BHQ1 | MSC_0361 | RmuC DNA recombinase (1 TMD) | -8,16 | 7 | 41 | 356 | 3 |  | 3 |  | 3 | 14 | 0,21 | ++ |
| A0A0F2BG35 | MSC_0730 | 50S ribosomal protein L6 | -7,03 | 12 | 20 | 180 | 2 |  | 2 |  | 2 | 10 | 0,20 | ++ |
| A0A0F2BHW4 | MSC_0300 | hypothetical ribonuclease J | -12,01 | 5 | 66 | 583 | 5 |  | 4 |  | 4 | 25 | 0,20 | ++ |
| A0A0F2BQG0 | MSC_0431 | predicted lipoprotein DUF285 | -8,14 | 9 | 40 | 353 | 3 |  | 3 |  | 3 | 16 | 0,19 | ++ |
| A0A0F2BGI4 | MSC_0114 | threonine dehydratase | -12,92 | 7 | 45 | 408 | 3 |  | 2 |  | 2 | 16 | 0,19 | ++ |
| A0A0F2BJ71 | MSC_0150 | Peptide chain release factor 1 | -10,24 | 9 | 41 | 364 | 3 |  | 3 |  | 3 | 17 | 0,18 | ++ |
| A0A0F2BKF7 | MSC_0971/978 | UDP glucose 4 epimerase | -19,43 | 10 | 38 | 334 | 3 |  | 3 |  | 3 | 18 | 0,17 | ++ |
| A0A0F2BKF8 | MSC_0260 | 6-phosphofructokinase | -10,02 | 6 | 35 | 326 | 2 |  | 2 |  | 2 | 12 | 0,17 | ++ |
| A0A126SSQ4 | MSC_0301 | oxydireductase | -4,49 | 7 | 26 | 239 | 2 |  | 2 |  | 2 | 12 | 0,17 | ++ |
| A0A0F2BK61 | MSC_0962 | Transcription antitermination protein NusG | -4,86 | 10 | 24 | 213 | 2 |  | 2 |  | 2 | 12 | 0,17 | ++ |
| A0A126STR6 | MSC_0577 | **1-acyl-sn-glycerol-3-phosphate acyltransferase** | -3,44 | 6 | 37 | 315 | 2 |  | 2 |  | 2 | 12 | 0,17 | ++ |
| A0A140DZP1 | MSC_0984 | UDP galactpyranose mutase | -11,84 | 8 | 46 | 395 | 3 |  | 3 |  | 3 | 19 | 0,16 | ++ |
| A0A0F2BK69 | MSC_0725 | adenylate kinase | -4,89 | 8 | 24 | 213 | 2 |  | 2 |  | 2 | 13 | 0,15 | ++ |
| A0A126SU45 | MSC_0422 | ribonuclease Y (1 TMD) | -8,45 | 5 | 57 | 509 | 3 |  | 3 |  | 3 | 22 | 0,14 | ++ |
| A0A0F2BHI5 | MSC_0509 | NADP-dependent glyceraldehyde-3-phosphate dehydrogenase | -14,05 | 9 | 52 | 471 | 4 |  | 4 |  | 4 | 30 | 0,13 | ++ |
| A0A126SRH4 | MSC_0481 | putative dihydroxyacetone kinase | -4,36 | 5 | 61 | 547 | 3 |  | 3 |  | 3 | 23 | 0,13 | ++ |
| A0A0F2BR16 | MSC_0269 | phosphate acyltransferase | -4,90 | 4 | 36 | 322 | 2 |  | 2 |  | 2 | 16 | 0,13 | ++ |
| A0A0F2BGA6 | MSC_0110/990 | UTP-glucose-1-phosphate uridylyltransferase | -4,51 | 11 | 33 | 290 | 2 |  | 2 |  | 2 | 16 | 0,13 | ++ |
| A0A140DZH3 | MSC_0110/990 | UTP-glucose-1-phosphate uridylyltransferase | -4,51 | 11 | 33 | 290 | 2 |  | 2 |  | 2 | 16 | 0,13 | ++ |
| A0A0F2BH47 | MSC_0721 | **DNA-directed RNA polymerase alpha chain** | -8,06 | 5 | 35 | 317 | 2 |  | 2 |  | 2 | 16 | 0,13 | ++ |
| A0A126SQS4 | MSC_0825 | phosphoglycerate mutase | -6,39 | 3 | 60 | 531 | 3 |  | 2 |  | 3 | 25 | 0,12 | ++ |
| A0A0F2BP18 | MSC_0829 | phosphoglucomutase | -11,39 | 6 | 64 | 558 | 3 |  | 3 |  | 3 | 25 | 0,12 | ++ |
| A0A126SSB6 | MSC_0267 | Dihydrolipoamide acetyltransferase (pyruvate dehydrogenase complex) | -18,01 | 8 | 46 | 428 | 2 |  | 2 |  | 2 | 17 | 0,12 | ++ |
| A0A140DZF6 | MSC_0103/0104 | predicted lipoprotein | -6,37 | 5 | 42 | 372 | 2 |  | 2 |  | 2 | 18 | 0,11 | ++ |
| A0A126SS16 | MSC_0253 | elonase | -13,17 | 5 | 49 | 451 | 2 |  | 2 |  | 2 | 19 | 0,11 | ++ |
| A0A0F2BHV4 | MSC_0619 | ATP synthase alpha chain | -10,33 | 3 | 58 | 515 | 2 |  | 2 |  | 2 | 20 | 0,10 | ++ |
| A0A109WHL4 | MSC_0013 | lipoprotein LppA P72 | -8,68 | 3 | 63 | 548 | 2 |  | 2 |  | 2 | 20 | 0,10 | ++ |
| A0A0F2BPB5 | MSC_0273 | PtsI PEP phosphotransferase | -9,64 | 3 | 64 | 573 | 2 |  | 2 |  | 2 | 21 | 0,10 | ++ |
| A0A140DZB7 | MSC_0066 | seryl-tRNA synthetase | -12,27 | 5 | 49 | 422 | 2 |  | 2 |  | 2 | 21 | 0,10 | ++ |
| A0A0F2BGK7 | MSC_0159 | elongation factor G | -14,48 | 4 | 76 | 689 | 3 |  | 3 |  | 3 | 33 | 0,09 | + |
| A0A140DZJ6 | MSC_1021/1046 | predicted liporotein DUF285 | -12,11 | 3 | 52 | 445 | 2 |  | 2 |  | 2 | 22 | 0,09 | + |
| A0A0F2BLT6 | MSC_0423 | signal recognition particle M54 | -7,47 | 3 | 50 | 447 | 2 |  | 2 |  | 2 | 23 | 0,09 | + |
| A0A0F2BHS9 | MSC_0775 | predicted lipoprotein | -7,27 | 2 | 83 | 727 | 2 |  | 2 |  | 2 | 30 | 0,07 | + |
| A0A126SRW4 | MSC_0333 | aspartyl-tRNA synthetase | -10,18 | 3 | 67 | 574 | 2 |  | 2 |  | 2 | 30 | 0,07 | + |
| A0A0F2BGX7 | MSC_0372 | transketolase | -7,32 | 3 | 73 | 656 | 2 |  | 2 |  | 2 | 32 | 0,06 | + |
| A0A126STV6 | MSC_0539 | hypothetical protein | -19,48 | 4 | 71 | 608 | 2 |  | 2 |  | 2 | 33 | 0,06 | + |
| A0A0F2BKF9 | MSC_1017 | tRNA uridine 5-carboxymethylaminomethyl modification enzyme | -8,46 | 3 | 71 | 629 | 2 |  | 2 |  | 2 | 33 | 0,06 | + |
| A0A126SR84 | MSC_0776 | predicted lipoprotein | -6,35 | 2 | 91 | 782 | 2 |  | 2 |  | 2 | 37 | 0,05 | + |
| A0A0F2BMU5 | MSC_0627 | predicted lipoptotein (DUF31 domain) | -13,88 | 3 | 97 | 851 | 2 |  | 2 |  | 2 | 44 | 0,05 | + |
| A0A0F2BPA4 | MSC_0500 | predicted lipoprotein | -8,91 | 2 | 108 | 970 | 2 |  | 2 |  | 2 | 46 | 0,04 | + |
| A0A126SRX3 | MSC_0457 | FtsX-like permease family (TMD, 2 ftsX domains) | -6,17 | 1 | 201 | 1754 | 2 |  | 2 |  | 2 | 76 | 0,03 | + |

| lipoproteins and transmembrane proteins |
| --- |
| membrane associated proteins |
| cytoplasmic proteins |
| PAI semi quantitative scale: ++++: ≥1.0; +++: [0.5-1.0[; ++: [0.1-0.5[; + <0.1. |
| Proteins retrieved in EV from the three species are in bold |

|  | **membrane** | **membrane-bound** | **cytoplasmic** |
| --- | --- | --- | --- |
| ***M. mycoides* subsp. *mycoides* Afadé EV** | **26,00** | **21** | **41** |
| **88 proteins** |  |  |  |
| **percentage** | **29,55** | **24** | **47** |


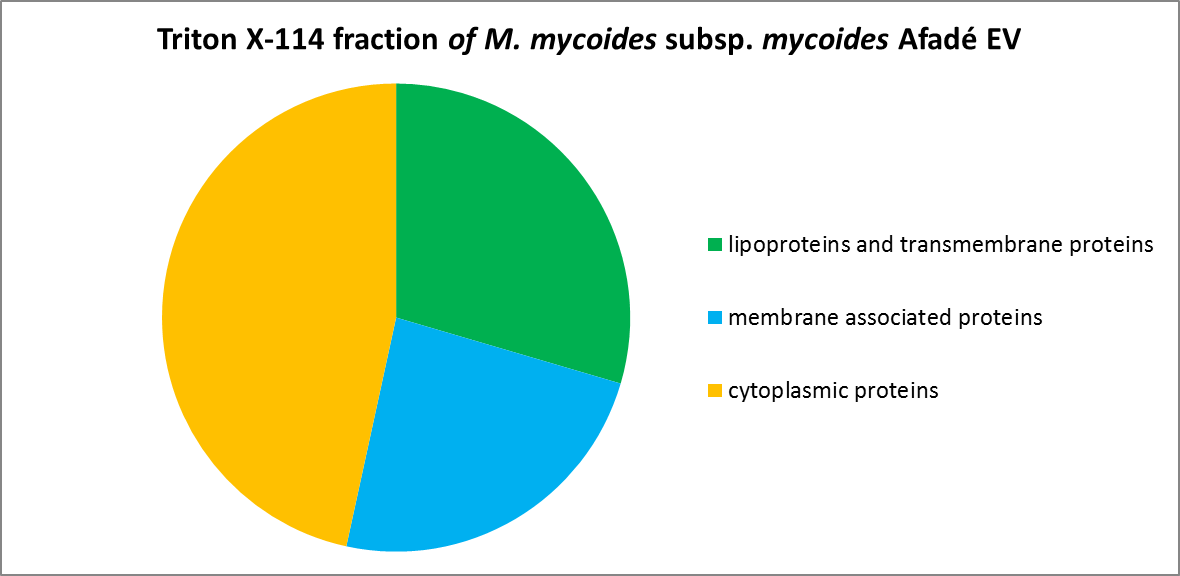


***M. fermentans* PG18^T^ (Triton X-114 fraction)**

| **Uniprot accession number** | **molligen accession number** | **predicted function** | **log(E value)** | **Coverage** | **MW** | **size** | **Spectra** | **Specific** | **Uniques** | **Specific uniques** | **Uniques peptide-mod-charge** | **Theoretical number of tryptic peptides** | **PAI** |  |
| --- | --- | --- | --- | --- | --- | --- | --- | --- | --- | --- | --- | --- | --- | --- |
| C4XE63 | MBIO_0170 | **Chaperone protein DnaK** | -429,19 | 59 | 64 | 585 | 106 |  | 50 |  | 70 | 21 | 5,05 | ++++ |
| C4XEI5 | MBIO_0292 | **elongation factor TU** | -463,50 | 71 | 48 | 434 | 97 | 7 | 53 | 4 | 71 | 22 | 4,41 | ++++ |
| C4XFE4 | MBIO_0601 | **lipoprotein p37** | -150,66 | 61 | 28 | 241 | 52 |  | 20 |  | 35 | 13 | 4,00 | ++++ |
| C4XDU3 | MBIO_0050 | chromosome seggregation ATPase | -277,23 | 73 | 49 | 426 | 54 |  | 34 |  | 48 | 14 | 3,86 | ++++ |
| Q9RGX5 | MBIO_0763 | macrophage activating lipoprotein-404 precursor | -328,17 | 67 | 48 | 428 | 78 | 1 | 42 | 1 | 62 | 21 | 3,71 | ++++ |
| C4XEL2 | MBIO_0319 | oligopeptide ABC transporter oppA (lipoprotein) | -706,58 | 70 | 106 | 942 | 146 |  | 85 |  | 113 | 42 | 3,48 | ++++ |
| C4XFY0 | MBIO_0787 | hypothetical membrane protein (1 TMD) | -122,59 | 38 | 40 | 358 | 17 |  | 13 |  | 15 | 5 | 3,40 | ++++ |
| C4XER1 | MBIO_0368 | hypothetical membrane protein P80-like (2 TMD) | -624,88 | 66 | 85 | 769 | 116 |  | 67 |  | 95 | 35 | 3,31 | ++++ |
| Q49159 | MBIO_0661 | lipoprotein p29 | -168,16 | 74 | 27 | 244 | 41 |  | 28 |  | 35 | 13 | 3,15 | ++++ |
| C4XEV3 | MBIO_0410 | phosphoglycerate kinase | -280,32 | 82 | 44 | 401 | 51 |  | 36 |  | 47 | 21 | 2,43 | ++++ |
| C4XDV0 | MBIO_0057 | Phosphopentomutase | -144,08 | 52 | 44 | 395 | 38 |  | 22 |  | 32 | 16 | 2,38 | ++++ |
| C4XFT3 | MBIO_0740 | hypothetical membrane protein (1 TMD) | -110,53 | 45 | 30 | 266 | 23 |  | 18 |  | 23 | 11 | 2,09 | ++++ |
| C4XFZ1 | MBIO_0798 | 30S ribosomal protein S13 | -26,77 | 41 | 14 | 123 | 8 |  | 6 |  | 8 | 4 | 2,00 | ++++ |
| C4XFT5 | MBIO_0742 | hypothetical protein | -175,43 | 70 | 40 | 353 | 35 |  | 22 |  | 31 | 18 | 1,94 | ++++ |
| Q5QGL0 | MBIO_0017 | prophage-like protein MEM (1 TMD) | -113,93 | 51 | 25 | 215 | 19 |  | 18 |  | 19 | 10 | 1,90 | ++++ |
| C4XFV4 | MBIO_0761 | ABC transporter | -181,48 | 47 | 68 | 608 | 41 |  | 28 |  | 37 | 22 | 1,86 | ++++ |
| C4XFV0 | MBIO_0757 | variable surface lipoprotein p78 | -374,74 | 59 | 79 | 682 | 62 |  | 43 |  | 60 | 35 | 1,77 | ++++ |
| C4XF62 | MBIO_0519 | predicted lipoprotein | -126,88 | 69 | 27 | 241 | 24 |  | 21 |  | 24 | 14 | 1,71 | ++++ |
| C4XEK9 | MBIO_0316 | permease PTS glucose | -141,30 | 62 | 33 | 294 | 28 |  | 23 |  | 28 | 17 | 1,65 | ++++ |
| C4XF61 | MBIO_0518 | glyceraldehyde-3-phosphate dehydrogenase | -124,46 | 48 | 37 | 339 | 24 |  | 15 |  | 20 | 15 | 1,60 | ++++ |
| C4XFD1 | MBIO_0588 | hypothetical membrane protein (1 TMD) | -234,45 | 55 | 67 | 582 | 41 |  | 30 |  | 40 | 26 | 1,58 | ++++ |
| C4XER2 | MBIO_0369 | lipoprotein nuclease family MAG5040-like | -150,13 | 59 | 46 | 399 | 29 |  | 21 |  | 28 | 19 | 1,53 | ++++ |
| C4XFW7 | MBIO_0774 | hypothetical membrane protein (1 TMD) | -512,02 | 49 | 148 | 1274 | 92 |  | 69 |  | 90 | 61 | 1,51 | ++++ |
| C4XEA1 | MBIO_0208 | predicted lipoprotein | -42,28 | 34 | 14 | 117 | 7 |  | 6 |  | 7 | 5 | 1,40 | ++++ |
| C4XG16 | MBIO_0823 | 30S ribosomal protein S10 | -36,23 | 59 | 11 | 101 | 7 |  | 6 |  | 7 | 5 | 1,40 | ++++ |
| C4XEU2 | MBIO_0399 | hypothetical membrane protein (4 TMD) | -37,20 | 19 | 28 | 244 | 8 |  | 5 |  | 6 | 6 | 1,33 | ++++ |
| C4XFT4 | MBIO_0741 | hypothetical membrane protein (4 TMD) | -24,57 | 18 | 22 | 181 | 8 |  | 3 |  | 6 | 6 | 1,33 | ++++ |
| C4XEV0 | MBIO_0407 | Triosephosphate isomerase | -69,41 | 41 | 29 | 262 | 13 |  | 9 |  | 13 | 10 | 1,30 | ++++ |
| C4XFE5 | MBIO_0602 | Alkylphosphonate ABC transporter | -76,29 | 38 | 29 | 250 | 14 |  | 10 |  | 14 | 11 | 1,27 | ++++ |
| C4XE16 | MBIO_0123 | hypothetical membrane protein DUF3137 domain (2 TMD) | -81,78 | 42 | 42 | 365 | 16 |  | 12 |  | 15 | 13 | 1,23 | ++++ |
| C4XG62 | MBIO_0869 | predicted lipoprotein | -205,18 | 42 | 88 | 792 | 43 |  | 34 |  | 42 | 35 | 1,23 | ++++ |
| C4XEN1 | MBIO_0338 | predicted lipoprotein | -192,38 | 53 | 65 | 569 | 32 |  | 29 |  | 31 | 27 | 1,19 | ++++ |
| C4XEC7 | MBIO_0234 | hypothetical membrane protein (1 TMD) | -173,35 | 43 | 57 | 493 | 26 |  | 21 |  | 26 | 22 | 1,18 | ++++ |
| C4XEX3 | MBIO_0430 | Deoxyadenosine kinase | -64,62 | 54 | 28 | 230 | 13 |  | 11 |  | 13 | 11 | 1,18 | ++++ |
| C4XG83 | MBIO_0890 | hypothetical membrane protein (1 TMD) | -375,50 | 49 | 148 | 1278 | 69 | 35 | 58 | 29 | 67 | 60 | 1,15 | ++++ |
| C4XEW1 | MBIO_0418 | hypothetical membrane protein (2 TMD) | -73,52 | 32 | 51 | 437 | 17 |  | 14 |  | 17 | 15 | 1,13 | ++++ |
| C4XEM3 | MBIO_0330 | Phosphoglycerate mutase | -83,16 | 32 | 57 | 507 | 18 |  | 15 |  | 18 | 16 | 1,13 | ++++ |
| C4XEC6 | MBIO_0233 | Pyruvate kinase | -98,34 | 41 | 53 | 477 | 19 |  | 18 |  | 19 | 17 | 1,12 | ++++ |
| C4XFZ8 | MBIO_0805 | 30S ribosomal protein S5 | -43,32 | 37 | 26 | 239 | 11 |  | 8 |  | 11 | 10 | 1,10 | ++++ |
| C4XE71 | MBIO_0178 | Chaperone protein DnaJ | -100,76 | 45 | 42 | 373 | 17 |  | 14 |  | 17 | 16 | 1,06 | ++++ |
| C4XDX7 | MBIO_0084 | 30S ribosomal protein S12 | -14,10 | 18 | 15 | 139 | 3 |  | 3 |  | 3 | 3 | 1,00 | ++++ |
| C4XDX8 | MBIO_0085 | 30S ribosomal protein S7 | -38,96 | 56 | 18 | 156 | 7 |  | 6 |  | 7 | 7 | 1,00 | ++++ |
| C4XEB8 | MBIO_0225 | 30S ribosomal protein S9 | -15,60 | 26 | 15 | 139 | 2 |  | 2 |  | 2 | 2 | 1,00 | ++++ |
| C4XEF7 | MBIO_0264 | predicted lipoprotein | -6,53 | 11 | 31 | 279 | 2 |  | 2 |  | 2 | 2 | 1,00 | ++++ |
| C4XEM5 | MBIO_0332 | hypothetical membrane protein (3 TMD) | -14,22 | 11 | 39 | 343 | 3 |  | 2 |  | 2 | 3 | 1,00 | ++++ |
| C4XEN0 | MBIO_0337 | hypothetical protein | -86,49 | 49 | 37 | 322 | 16 |  | 14 |  | 15 | 16 | 1,00 | ++++ |
| C4XEY1 | MBIO_0438 | amino acid permease | -48,19 | 10 | 65 | 591 | 6 |  | 5 |  | 6 | 6 | 1,00 | ++++ |
| C4XFC6 | MBIO_0583 | hypothetical membrane protein (3 TMD) | -6,03 | 12 | 16 | 139 | 3 |  | 2 |  | 2 | 3 | 1,00 | ++++ |
| C4XFJ0 | MBIO_0647 | lipoprotein BspA-like leucine rich repat protein | -51,13 | 40 | 28 | 248 | 9 |  | 9 |  | 9 | 9 | 1,00 | ++++ |
| C4XFK9 | MBIO_0666 | YebC/PmpR family regulator | -24,32 | 14 | 28 | 251 | 5 |  | 4 |  | 5 | 5 | 1,00 | ++++ |
| C4XFN7 | MBIO_0694 | hypothetical membrane protein (3 TMD) | -9,95 | 20 | 14 | 128 | 2 |  | 2 |  | 2 | 2 | 1,00 | ++++ |
| C4XDX9 | MBIO_0086 | Translation elongation factor G | -164,97 | 43 | 77 | 696 | 30 |  | 23 |  | 29 | 31 | 0,97 | +++ |
| O06762 | MBIO_0759 | ABC transporter | -49,54 | 14 | 62 | 536 | 13 |  | 12 |  | 13 | 14 | 0,93 | +++ |
| C4XER3 | MBIO_0370 | ABC transporter | -160,03 | 39 | 82 | 703 | 32 |  | 27 |  | 32 | 35 | 0,91 | +++ |
| C4XEX5 | MBIO_0432 | hypothetical membrane protein (1 TMD) | -36,03 | 32 | 28 | 242 | 10 |  | 9 |  | 9 | 11 | 0,91 | +++ |
| C4XE89 | MBIO_0196 | trigger factor (prolyl isomerase) | -98,61 | 32 | 59 | 511 | 18 |  | 17 |  | 18 | 20 | 0,90 | +++ |
| C4XEL6 | MBIO_0323 | oligopeptide ABC transporter | -183,53 | 36 | 104 | 889 | 35 | 34 | 32 | 31 | 35 | 39 | 0,90 | +++ |
| C4XFT0 | MBIO_0737 | hypothetical membrane protein (4 TMD) | -22,38 | 21 | 37 | 322 | 6 |  | 6 |  | 6 | 7 | 0,86 | +++ |
| C4XFM0 | MBIO_0677 | lipoprotein | -241,57 | 43 | 106 | 932 | 42 |  | 37 |  | 41 | 52 | 0,81 | +++ |
| C4XFT9 | MBIO_0746 | hypothetical membrane protein (5 TMD) | -155,43 | 37 | 77 | 663 | 25 |  | 23 |  | 25 | 31 | 0,81 | +++ |
| C4XEX4 | MBIO_0431 | Transcription elongation factor GreA | -35,09 | 28 | 18 | 163 | 4 |  | 4 |  | 4 | 5 | 0,80 | +++ |
| C4XFS0 | MBIO_0727 | hypothetical membrane protein (1 TMD) | -41,30 | 39 | 24 | 197 | 8 |  | 7 |  | 8 | 10 | 0,80 | +++ |
| C4XEL5 | MBIO_0322 | oligopeptide ABC transporter oppD | -84,75 | 34 | 42 | 372 | 11 |  | 10 |  | 11 | 14 | 0,79 | +++ |
| C4XG31 | MBIO_0838 | hypothetical membrane protein (1 TMD) | -32,97 | 32 | 28 | 244 | 6 |  | 6 |  | 6 | 8 | 0,75 | +++ |
| C4XFD5 | MBIO_0592 | glucose 6P isomerase | -75,73 | 33 | 48 | 426 | 17 |  | 14 |  | 17 | 23 | 0,74 | +++ |
| C4XF05 | MBIO_0462 | elongation factor TS | -65,61 | 28 | 32 | 294 | 10 |  | 9 |  | 10 | 14 | 0,71 | +++ |
| C4XF76 | MBIO_0533 | lipoprotein P60-like | -99,89 | 37 | 51 | 445 | 15 |  | 15 |  | 15 | 21 | 0,71 | +++ |
| O06763 | MBIO_0758 | sugar ABC transporter | -24,60 | 10 | 36 | 326 | 5 |  | 3 |  | 5 | 7 | 0,71 | +++ |
| C4XFW4 | MBIO_0771 | permidine/putrescine ABC transporter | -47,96 | 27 | 37 | 323 | 10 |  | 9 |  | 10 | 14 | 0,71 | +++ |
| C4XDT4 | MBIO_0041 | predicted lipoprotein | -70,61 | 33 | 68 | 597 | 21 |  | 20 |  | 21 | 30 | 0,70 | +++ |
| C4XFB4 | MBIO_0571 | hypothetical membrane protein (1 TMD) | -203,86 | 29 | 147 | 1271 | 43 | 9 | 38 | 9 | 42 | 63 | 0,68 | +++ |
| C4XEX0 | MBIO_0427 | glucosamine-6-phosphate deaminase | -31,63 | 33 | 29 | 251 | 6 |  | 6 |  | 6 | 9 | 0,67 | +++ |
| C4XF82 | MBIO_0539 | hypothetical membrane protein (1 TMD) | -17,23 | 16 | 16 | 146 | 2 |  | 2 |  | 2 | 3 | 0,67 | +++ |
| C4XFD0 | MBIO_0587 | glycyl-tRNA synthetase | -91,99 | 37 | 54 | 466 | 16 |  | 15 |  | 16 | 24 | 0,67 | +++ |
| C4XFY8 | MBIO_0795 | 50S ribosomal protein L17 | -11,57 | 15 | 18 | 158 | 2 |  | 2 |  | 2 | 3 | 0,67 | +++ |
| C4XG01 | MBIO_0808 | 30S ribosomal protein S8 | -27,70 | 45 | 15 | 134 | 4 |  | 4 |  | 4 | 6 | 0,67 | +++ |
| C4XER7 | MBIO_0374 | lipoprotein | -96,81 | 36 | 59 | 502 | 17 |  | 16 |  | 17 | 26 | 0,65 | +++ |
| C4XEL4 | MBIO_0321 | ABC transporter oligopeptide oppC | -54,37 | 28 | 45 | 403 | 11 |  | 9 |  | 11 | 17 | 0,65 | +++ |
| C4XFY4 | MBIO_0790 | COF family HAD hydrolase | -44,10 | 32 | 33 | 292 | 7 |  | 6 |  | 7 | 11 | 0,64 | +++ |
| C4XEL3 | MBIO_0320 | ABC transporter oligopeptide oppB | -35,25 | 15 | 42 | 365 | 5 |  | 4 |  | 5 | 8 | 0,63 | +++ |
| C4XFI8 | MBIO_0645 | Lipoate-protein ligase A | -38,36 | 26 | 38 | 336 | 10 |  | 9 |  | 10 | 16 | 0,63 | +++ |
| C4XF52 | MBIO_0509 | serine/threonine protein kinase (1TMD) | -46,67 | 29 | 38 | 332 | 8 |  | 8 |  | 8 | 13 | 0,62 | +++ |
| C4XF89 | MBIO_0546 | hypothetical membrane protein (1 TMD) | -90,24 | 30 | 82 | 696 | 19 |  | 18 |  | 18 | 31 | 0,61 | +++ |
| C4XEL0 | MBIO_0317 | D-lactate dehydrogenase | -75,70 | 39 | 39 | 350 | 11 |  | 11 |  | 11 | 18 | 0,61 | +++ |
| C4XDV8 | MBIO_0065 | hypothetical membrane protein (3 TMD) | -6,35 | 15 | 22 | 187 | 3 |  | 3 |  | 3 | 5 | 0,60 | +++ |
| C4XEY0 | MBIO_0437 | predicted lipoprotein | -13,58 | 23 | 15 | 127 | 3 |  | 3 |  | 3 | 5 | 0,60 | +++ |
| C4XFI1 | MBIO_0638 | hypothetical protein | -28,98 | 38 | 19 | 164 | 6 |  | 5 |  | 6 | 10 | 0,60 | +++ |
| C4XFS3 | MBIO_0730 | predicted lipoprotein | -36,56 | 33 | 25 | 214 | 6 |  | 6 |  | 6 | 10 | 0,60 | +++ |
| C4XG71 | MBIO_0878 | 50S ribosomal protein L27 | -14,74 | 26 | 14 | 132 | 3 |  | 3 |  | 3 | 5 | 0,60 | +++ |
| C4XG09 | MBIO_0816 | 30S ribosomal protein S3 | -31,46 | 26 | 25 | 225 | 7 |  | 7 |  | 7 | 12 | 0,58 | +++ |
| C4XEK0 | MBIO_0307 | ATP synthase alpha chain | -53,88 | 21 | 58 | 528 | 10 |  | 10 |  | 10 | 18 | 0,56 | +++ |
| C4XG12 | MBIO_0819 | **50S ribosomal protein L2** | -31,60 | 24 | 31 | 283 | 5 |  | 5 |  | 5 | 9 | 0,56 | +++ |
| C4XFN0 | MBIO_0687 | Cell division protein FtsH (2 TMD) | -122,48 | 23 | 78 | 708 | 16 |  | 14 |  | 16 | 29 | 0,55 | +++ |
| C4XE74 | MBIO_0181 | **1-acyl-sn-glycerol-3-phosphateacyl transferase PlsC** | -24,52 | 25 | 30 | 257 | 7 |  | 6 |  | 7 | 13 | 0,54 | +++ |
| C4XF53 | MBIO_0510 | phosphatase | -33,84 | 36 | 29 | 260 | 7 |  | 7 |  | 7 | 13 | 0,54 | +++ |
| C4XDX0 | MBIO_0077 | predicted lipoprotein | -108,19 | 33 | 92 | 784 | 23 |  | 21 |  | 22 | 43 | 0,53 | +++ |
| C4XE31 | MBIO_0138 | InfB Translation initiation factor 2 | -93,26 | 30 | 66 | 601 | 15 |  | 13 |  | 15 | 30 | 0,50 | +++ |
| C4XE57 | MBIO_0164 | hypothetical membrane protein (4 TMD) | -77,19 | 21 | 55 | 487 | 9 |  | 9 |  | 9 | 18 | 0,50 | +++ |
| C4XEP3 | MBIO_0350 | Single-stranded DNA-binding protein | -12,79 | 15 | 20 | 179 | 2 |  | 2 |  | 2 | 4 | 0,50 | +++ |
| C4XES8 | MBIO_0385 | Heat shock protein GrpE | -49,98 | 22 | 48 | 418 | 8 |  | 8 |  | 8 | 16 | 0,50 | +++ |
| C4XES9 | MBIO_0386 | mechanosensitive ion channel | -10,32 | 20 | 15 | 139 | 2 |  | 2 |  | 2 | 4 | 0,50 | +++ |
| C4XEV4 | MBIO_0411 | PTS glucose permease | -17,08 | 26 | 16 | 137 | 3 |  | 2 |  | 3 | 6 | 0,50 | +++ |
| C4XF17 | MBIO_0474 | hypothetical membrane protein (1 TMD) | -21,07 | 33 | 22 | 193 | 5 |  | 5 |  | 5 | 10 | 0,50 | +++ |
| C4XFS1 | MBIO_0728 | potassium channel | -24,33 | 4 | 45 | 391 | 5 |  | 3 |  | 5 | 10 | 0,50 | +++ |
| C4XF77 | MBIO_0534 | hypothetical membrane protein (1 TMD) | -100,28 | 34 | 85 | 735 | 18 |  | 18 |  | 18 | 37 | 0,49 | ++ |
| C4XEJ3 | MBIO_0300 | Signal recognition particle, subunit Ffh SRP54 | -47,75 | 25 | 51 | 459 | 11 |  | 10 |  | 11 | 23 | 0,48 | ++ |
| C4XFU2 | MBIO_0749 | Seryl-tRNA synthetase | -35,68 | 28 | 48 | 423 | 10 |  | 8 |  | 8 | 21 | 0,48 | ++ |
| C4XF16 | MBIO_0473 | Mg/Co/Ni transporter MgtE | -40,43 | 20 | 54 | 480 | 6 |  | 6 |  | 6 | 13 | 0,46 | ++ |
| C4XE69 | MBIO_0176 | Serine hydroxymethyltransferase | -54,29 | 30 | 47 | 424 | 10 |  | 9 |  | 10 | 22 | 0,45 | ++ |
| Q9RFP7 | MBIO_0636 | hypothetical protein | -19,02 | 17 | 27 | 232 | 5 |  | 5 |  | 5 | 11 | 0,45 | ++ |
| C4XEC4 | MBIO_0231 | Fructose-bisphosphate aldolase | -31,77 | 22 | 31 | 287 | 4 |  | 4 |  | 4 | 9 | 0,44 | ++ |
| C4XEX1 | MBIO_0428 | Hypoxanthine-guanine phosphoribosyltransferase | -23,98 | 21 | 22 | 196 | 4 |  | 4 |  | 4 | 9 | 0,44 | ++ |
| C4XFQ8 | MBIO_0715 | ATP-dependent ClpB protein | -88,94 | 26 | 84 | 741 | 16 |  | 16 |  | 16 | 36 | 0,44 | ++ |
| C4XFK0 | MBIO_0657 | Ascorbate-specific PTS system, EIIC component | -43,62 | 10 | 77 | 712 | 7 |  | 5 |  | 7 | 16 | 0,44 | ++ |
| C4XFW3 | MBIO_0770 | DNA polymerase III subunits gamma and tau | -78,42 | 24 | 70 | 612 | 14 |  | 13 |  | 14 | 32 | 0,44 | ++ |
| C4XFT7 | MBIO_0744 | Transketolase | -89,50 | 28 | 73 | 648 | 13 |  | 13 |  | 13 | 30 | 0,43 | ++ |
| C4XEQ4 | MBIO_0361 | hypothetical protein | -24,51 | 20 | 33 | 290 | 6 |  | 5 |  | 6 | 14 | 0,43 | ++ |
| C4XF64 | MBIO_0521 | N-acetylglucosamine-specific IIC IIB component | -40,74 | 11 | 62 | 585 | 6 |  | 5 |  | 6 | 14 | 0,43 | ++ |
| C4XF80 | MBIO_0537 | Ribosome recycling factor | -15,55 | 15 | 21 | 183 | 3 |  | 2 |  | 3 | 7 | 0,43 | ++ |
| C4XED8 | MBIO_0245 | hypothetical membrane protein (1 TMD) | -26,57 | 12 | 37 | 328 | 5 |  | 3 |  | 5 | 12 | 0,42 | ++ |
| C4XES5 | MBIO_0382 | 30S ribosomal protein S4 | -36,61 | 24 | 25 | 216 | 5 |  | 5 |  | 5 | 12 | 0,42 | ++ |
| C4XET2 | MBIO_0389 | Translation initiation factor 3 | -31,83 | 25 | 25 | 218 | 5 |  | 5 |  | 5 | 12 | 0,42 | ++ |
| C4XFW6 | MBIO_0773 | Purine nucleoside phosphorylase | -31,26 | 21 | 27 | 236 | 5 |  | 5 |  | 5 | 12 | 0,42 | ++ |
| C4XEV7 | MBIO_0414 | Threonyl-tRNA synthetase | -69,55 | 26 | 68 | 582 | 13 |  | 13 |  | 13 | 32 | 0,41 | ++ |
| C4XFI9 | MBIO_0646 | N-acetylglucosamine-specific IIABC component | -65,25 | 17 | 97 | 885 | 13 |  | 12 |  | 13 | 32 | 0,41 | ++ |
| C4XEQ7 | MBIO_0364 | Oligopeptide transport ATP-binding protein OppF | -86,29 | 18 | 102 | 863 | 17 | 16 | 15 | 14 | 17 | 42 | 0,40 | ++ |
| C4XE47 | MBIO_0154 | Signal recognition particle receptor protein FtsY (membrane) | -28,94 | 17 | 40 | 355 | 6 |  | 6 |  | 6 | 15 | 0,40 | ++ |
| C4XEK2 | MBIO_0309 | ATP synthase beta chain | -66,66 | 21 | 55 | 502 | 8 |  | 8 |  | 8 | 20 | 0,40 | ++ |
| C4XEP2 | MBIO_0349 | 30S ribosomal protein S6 | -9,76 | 12 | 18 | 157 | 2 |  | 2 |  | 2 | 5 | 0,40 | ++ |
| C4XEW9 | MBIO_0426 | GTPase ObgE | -48,91 | 22 | 47 | 426 | 10 |  | 9 |  | 10 | 25 | 0,40 | ++ |
| C4XFZ4 | MBIO_0801 | Adenylate kinase | -41,75 | 34 | 26 | 228 | 6 |  | 6 |  | 6 | 15 | 0,40 | ++ |
| C4XG03 | MBIO_0810 | 50S ribosomal protein L5 | -37,07 | 28 | 21 | 183 | 4 |  | 4 |  | 4 | 10 | 0,40 | ++ |
| C4XFK7 | MBIO_0664 | Fructose-6-phosphate phosphoketolase | -73,44 | 18 | 90 | 792 | 13 |  | 11 |  | 13 | 33 | 0,39 | ++ |
| Q8GCK7 | MBIO_0560 | lipoprotein p57 | -38,42 | 17 | 62 | 522 | 9 |  | 8 |  | 9 | 23 | 0,39 | ++ |
| C4XDP4 | MBIO_0001 | putrescine transport ATP-binding protein PotA | -39,55 | 18 | 55 | 465 | 7 |  | 6 |  | 7 | 18 | 0,39 | ++ |
| C4XF04 | MBIO_0461 | 30S ribosomal protein S2 | -38,27 | 27 | 38 | 330 | 7 |  | 7 |  | 7 | 18 | 0,39 | ++ |
| C4XDR6 | MBIO_0023 | Type I restriction-modification system, DNA-methyltransferase | -58,59 | 28 | 59 | 503 | 10 |  | 10 |  | 10 | 26 | 0,38 | ++ |
| C4XE61 | MBIO_0168 | HAD hydrolase | -24,96 | 20 | 35 | 310 | 5 |  | 5 |  | 5 | 13 | 0,38 | ++ |
| C4XEY6 | MBIO_0443 | hypothetical protein | -45,29 | 28 | 35 | 304 | 8 |  | 7 |  | 8 | 21 | 0,38 | ++ |
| C4XFN6 | MBIO_0693 | 50S ribosomal protein L11 | -21,32 | 20 | 22 | 202 | 3 |  | 3 |  | 3 | 8 | 0,38 | ++ |
| C4XG36 | MBIO_0843 | hypothetical protein | -31,88 | 25 | 34 | 297 | 6 |  | 6 |  | 6 | 16 | 0,38 | ++ |
| Q9X495 | MBIO_0764 | hypothetical protein | -48,00 | 23 | 62 | 550 | 10 |  | 10 |  | 10 | 27 | 0,37 | ++ |
| C4XDU6 | MBIO_0053 | putative dihydroxyacetone kinase | -30,35 | 18 | 59 | 541 | 7 |  | 7 |  | 7 | 19 | 0,37 | ++ |
| C4XER8 | MBIO_0375 | hypothetical membrane protein (1 TMD) | -40,54 | 15 | 50 | 435 | 7 |  | 6 |  | 7 | 19 | 0,37 | ++ |
| C4XDZ7 | MBIO_0104 | hypothetical membrane protein (5 TMD) | -75,96 | 17 | 83 | 708 | 11 |  | 11 |  | 11 | 30 | 0,37 | ++ |
| C4XDV9 | MBIO_0066 | Copper-translocating P-type ATPase (10 TMD) | -98,29 | 20 | 99 | 900 | 12 |  | 11 |  | 12 | 33 | 0,36 | ++ |
| C4XEY8 | MBIO_0445 | PTS enzyme I | -69,54 | 23 | 67 | 593 | 10 |  | 9 |  | 10 | 28 | 0,36 | ++ |
| C4XG15 | MBIO_0822 | 50S ribosomal protein L3 | -16,39 | 20 | 30 | 276 | 5 |  | 5 |  | 5 | 14 | 0,36 | ++ |
| C4XDY6 | MBIO_0093 | 50S ribosomal protein L7/L12 | -7,42 | 18 | 13 | 123 | 2 |  | 2 |  | 2 | 6 | 0,33 | ++ |
| C4XDZ1 | MBIO_0098 | hypothetical membrane protein (2 TMD) | -45,91 | 14 | 74 | 656 | 9 |  | 9 |  | 9 | 27 | 0,33 | ++ |
| C4XE42 | MBIO_0149 | 30S ribosomal protein S16 | -10,26 | 13 | 19 | 171 | 2 |  | 2 |  | 2 | 6 | 0,33 | ++ |
| C4XF08 | MBIO_0465 | hypothetical protein | -33,51 | 21 | 35 | 301 | 4 |  | 4 |  | 4 | 12 | 0,33 | ++ |
| C4XFN8 | MBIO_0695 | ribonuclease J | -65,65 | 21 | 72 | 644 | 11 |  | 11 |  | 11 | 33 | 0,33 | ++ |
| C4XFZ7 | MBIO_0804 | 50S ribosomal protein L15 | -11,59 | 14 | 17 | 154 | 2 |  | 2 |  | 2 | 6 | 0,33 | ++ |
| C4XEH1 | MBIO_0278 | lipoprotein | -46,96 | 18 | 60 | 510 | 8 |  | 7 |  | 8 | 25 | 0,32 | ++ |
| C4XDV7 | MBIO_0064 | Ribosome-binding ATPase | -29,20 | 19 | 41 | 366 | 6 |  | 6 |  | 6 | 19 | 0,32 | ++ |
| C4XEA3 | MBIO_0210 | GTP-binding protein | -19,95 | 13 | 50 | 435 | 6 |  | 5 |  | 6 | 19 | 0,32 | ++ |
| C4XEA4 | MBIO_0211 | Cytidylate kinase | -22,95 | 23 | 26 | 228 | 4 |  | 4 |  | 4 | 13 | 0,31 | ++ |
| C4XED1 | MBIO_0238 | Phosphate acetyltransferase | -32,31 | 22 | 35 | 317 | 4 |  | 4 |  | 4 | 13 | 0,31 | ++ |
| C4XEW5 | MBIO_0422 | 6-phosphofructokinase | -12,75 | 10 | 37 | 338 | 4 |  | 4 |  | 4 | 13 | 0,31 | ++ |
| C4XF26 | MBIO_0483 | membrane protein (1 TMD) | -15,52 | 15 | 35 | 306 | 4 |  | 4 |  | 4 | 13 | 0,31 | ++ |
| C4XFP3 | MBIO_0700 | Hydrolase of the HAD superfamily | -8,58 | 12 | 32 | 284 | 4 |  | 3 |  | 3 | 13 | 0,31 | ++ |
| C4XF55 | MBIO_0512 | hypotheticla membrane protein (2 TMD) | -34,73 | 16 | 51 | 436 | 6 |  | 6 |  | 6 | 20 | 0,30 | ++ |
| C4XE19 | MBIO_0126 | XAA-PRO aminopeptidase | -20,09 | 15 | 42 | 365 | 5 |  | 5 |  | 5 | 17 | 0,29 | ++ |
| C4XEU8 | MBIO_0405 | predicted lipoprotein | -43,20 | 20 | 43 | 371 | 5 |  | 5 |  | 5 | 17 | 0,29 | ++ |
| C4XE33 | MBIO_0140 | Transcription termination protein NusA | -39,37 | 20 | 61 | 538 | 7 |  | 7 |  | 7 | 24 | 0,29 | ++ |
| C4XDY9 | MBIO_0096 | Replicative DNA helicase | -27,84 | 17 | 54 | 474 | 6 |  | 6 |  | 6 | 21 | 0,29 | ++ |
| C4XE97 | MBIO_0204 | hypothetical membrane protein (5 TMD) | -33,25 | 14 | 62 | 548 | 6 |  | 6 |  | 6 | 21 | 0,29 | ++ |
| C4XFI0 | MBIO_0637 | hypothetical protein | -8,58 | 13 | 21 | 184 | 2 |  | 2 |  | 2 | 7 | 0,29 | ++ |
| C4XFU9 | MBIO_0756 | Thioredoxin | -18,86 | 28 | 12 | 104 | 2 |  | 2 |  | 2 | 7 | 0,29 | ++ |
| C4XFX0 | MBIO_0777 | Peptide chain release factor 1 | -36,26 | 20 | 42 | 373 | 7 |  | 7 |  | 7 | 25 | 0,28 | ++ |
| C4XFG7 | MBIO_0624 | Isoleucyl-tRNA synthetase | -56,74 | 17 | 103 | 893 | 12 |  | 12 |  | 12 | 43 | 0,28 | ++ |
| C4XED5 | MBIO_0242 | Methenyltetrahydrofolate cyclohydrolase | -11,99 | 11 | 33 | 298 | 3 |  | 3 |  | 3 | 11 | 0,27 | ++ |
| C4XFX3 | MBIO_0780 | Transcription antitermination protein NusG | -6,72 | 12 | 24 | 205 | 3 |  | 3 |  | 3 | 11 | 0,27 | ++ |
| C4XG14 | MBIO_0821 | 50S ribosomal protein L4 | -13,81 | 6 | 41 | 378 | 3 |  | 3 |  | 3 | 11 | 0,27 | ++ |
| C4XEI0 | MBIO_0287 | hypothetical protein | -27,75 | 21 | 27 | 235 | 4 |  | 3 |  | 4 | 15 | 0,27 | ++ |
| C4XEP1 | MBIO_0348 | DNA topoisomerase I | -61,66 | 16 | 72 | 629 | 8 |  | 8 |  | 8 | 30 | 0,27 | ++ |
| C4XEM6 | MBIO_0333 | Preprotein translocase SecA | -65,05 | 19 | 97 | 841 | 13 |  | 13 |  | 13 | 49 | 0,27 | ++ |
| C4XEN2 | MBIO_0339 | membrane ribonuclease (1 TMD) | -39,60 | 18 | 57 | 508 | 7 |  | 7 |  | 7 | 27 | 0,26 | ++ |
| C4XDU2 | MBIO_0049 | hypothetical membrane protein (5 TMD) | -9,14 | 7 | 35 | 302 | 2 |  | 2 |  | 2 | 8 | 0,25 | ++ |
| C4XE70 | MBIO_0177 | MatE efflux permease | -18,70 | 8 | 69 | 612 | 4 |  | 4 |  | 4 | 16 | 0,25 | ++ |
| C4XEA0 | MBIO_0207 | Thiol peroxidase | -9,68 | 8 | 19 | 169 | 2 |  | 2 |  | 2 | 8 | 0,25 | ++ |
| C4XED0 | MBIO_0237 | Acetate kinase | -15,57 | 10 | 44 | 398 | 4 |  | 4 |  | 4 | 16 | 0,25 | ++ |
| C4XEP4 | MBIO_0351 | 30S ribosomal protein S18 | -12,18 | 33 | 12 | 97 | 2 |  | 2 |  | 2 | 8 | 0,25 | ++ |
| C4XEW6 | MBIO_0423 | lipoprotein hydrolase HAD | -7,70 | 12 | 32 | 275 | 3 |  | 3 |  | 3 | 12 | 0,25 | ++ |
| Q6GYZ9 | MBIO_0496 | Thymidine kinase | -18,17 | 9 | 22 | 192 | 2 |  | 2 |  | 2 | 8 | 0,25 | ++ |
| C4XFS2 | MBIO_0729 | hypothetical membrane protein (1 TMD) | -30,79 | 14 | 77 | 654 | 9 |  | 8 |  | 8 | 38 | 0,24 | ++ |
| C4XEM2 | MBIO_0329 | predicted lipoprotein | -36,45 | 15 | 64 | 597 | 6 |  | 6 |  | 6 | 26 | 0,23 | ++ |
| C4XFN4 | MBIO_0691 | Cholinephosphate cytidylyltransferase | -49,40 | 15 | 70 | 608 | 6 |  | 6 |  | 6 | 26 | 0,23 | ++ |
| C4XFR3 | MBIO_0720 | DNA-methyltransferase subunit M | -20,66 | 14 | 64 | 560 | 6 |  | 6 |  | 6 | 26 | 0,23 | ++ |
| C4XFC1 | MBIO_0578 | ATP-dependent protease La (heat shock) | -67,16 | 12 | 116 | 1016 | 11 |  | 10 |  | 11 | 48 | 0,23 | ++ |
| C4XFL0 | MBIO_0667 | NAD synthetase | -26,26 | 16 | 30 | 270 | 3 |  | 3 |  | 3 | 14 | 0,21 | ++ |
| Q8GCM8 | MBIO_0549 | hypothetical protein | -39,56 | 13 | 83 | 700 | 7 |  | 7 |  | 7 | 34 | 0,21 | ++ |
| C4XDW7 | MBIO_0074 | sulfuryl transferase | -9,47 | 3 | 43 | 377 | 2 |  | 2 |  | 2 | 10 | 0,20 | ++ |
| C4XEQ6 | MBIO_0363 | hypothetical membrane protein (1 TMD) | -19,08 | 6 | 39 | 336 | 4 |  | 4 |  | 4 | 20 | 0,20 | ++ |
| C4XF46 | MBIO_0503 | Prolipoprotein diacylglyceryl transferase | -6,73 | 9 | 36 | 310 | 2 |  | 2 |  | 2 | 10 | 0,20 | ++ |
| C4XFI7 | MBIO_0644 | Esterase/lipase | -13,72 | 15 | 34 | 293 | 3 |  | 3 |  | 3 | 15 | 0,20 | ++ |
| C4XES3 | MBIO_0380 | DNA gyrase subunit A | -26,22 | 9 | 96 | 854 | 7 |  | 7 |  | 7 | 36 | 0,19 | ++ |
| C4XFU6 | MBIO_0753 | DNA gyrase subunit B | -43,32 | 15 | 72 | 644 | 6 |  | 6 |  | 6 | 31 | 0,19 | ++ |
| C4XF12 | MBIO_0469 | glycosyltransferase | -15,89 | 11 | 40 | 335 | 4 |  | 4 |  | 4 | 21 | 0,19 | ++ |
| C4XFN2 | MBIO_0689 | ABC transporter | -21,19 | 11 | 42 | 368 | 3 |  | 3 |  | 3 | 16 | 0,19 | ++ |
| C4XE99 | MBIO_0206 | Purine nucleoside phosphorylase | -5,79 | 10 | 26 | 237 | 2 |  | 2 |  | 2 | 11 | 0,18 | ++ |
| C4XEV1 | MBIO_0408 | Glucosamine-6-phosphate deaminase | -9,82 | 8 | 28 | 245 | 2 |  | 2 |  | 2 | 11 | 0,18 | ++ |
| C4XF54 | MBIO_0511 | Guanylate kinase | -8,35 | 11 | 25 | 212 | 2 |  | 2 |  | 2 | 11 | 0,18 | ++ |
| C4XFY5 | MBIO_0792 | Transmembrane component of ECF transporters (5 TMD) | -7,06 | 9 | 34 | 289 | 2 |  | 2 |  | 2 | 11 | 0,18 | ++ |
| C4XFU5 | MBIO_0752 | hypothetical membrane protein (6 TMD) | -21,64 | 8 | 56 | 495 | 3 |  | 3 |  | 3 | 17 | 0,18 | ++ |
| C4XEJ0 | MBIO_0297 | hypothetical membrane protein (1 TMD) | -29,84 | 11 | 77 | 659 | 6 |  | 6 |  | 6 | 35 | 0,17 | ++ |
| C4XEX7 | MBIO_0434 | Alanyl-tRNA synthetase | -44,22 | 10 | 100 | 875 | 7 |  | 7 |  | 7 | 41 | 0,17 | ++ |
| Q5QGL4 | MBIO_0014 | hypothetical protein | -10,03 | 8 | 37 | 330 | 2 |  | 2 |  | 2 | 12 | 0,17 | ++ |
| C4XDU5 | MBIO_0052 | fatty acid/phospholipid synthesis protein (PlsX) | -12,28 | 11 | 38 | 350 | 3 |  | 3 |  | 3 | 18 | 0,17 | ++ |
| C4XDV3 | MBIO_0060 | DNA polymerase III, delta subunit | -8,49 | 8 | 37 | 321 | 2 |  | 2 |  | 2 | 12 | 0,17 | ++ |
| C4XEN6 | MBIO_0343 | DNA-directed RNA polymerase beta chain | -56,11 | 12 | 136 | 1214 | 10 |  | 10 |  | 10 | 60 | 0,17 | ++ |
| C4XES7 | MBIO_0384 | Heat-inducible transcription repressor HrcA | -15,84 | 12 | 38 | 341 | 3 |  | 3 |  | 3 | 18 | 0,17 | ++ |
| C4XF45 | MBIO_0502 | hypotheticalr kinase/phosphorylase | -6,36 | 9 | 35 | 313 | 2 |  | 2 |  | 2 | 12 | 0,17 | ++ |
| C4XF84 | MBIO_0541 | DNA polymerase I | -8,81 | 9 | 34 | 300 | 2 |  | 2 |  | 2 | 12 | 0,17 | ++ |
| C4XF87 | MBIO_0544 | lipoprotein acid phosphatase | -22,40 | 12 | 41 | 367 | 3 |  | 3 |  | 3 | 18 | 0,17 | ++ |
| C4XFU4 | MBIO_0751 | Esterase/lipase | -9,98 | 10 | 36 | 310 | 3 |  | 3 |  | 3 | 18 | 0,17 | ++ |
| C4XG35 | MBIO_0842 | Glutamyl-tRNA(Gln) synthetase | -18,79 | 7 | 54 | 463 | 3 |  | 3 |  | 3 | 18 | 0,17 | ++ |
| Q8GCP5 | MBIO_0401 | Translation elongation factor LepA | -22,85 | 7 | 68 | 599 | 4 |  | 4 |  | 4 | 26 | 0,15 | ++ |
| C4XEW4 | MBIO_0421 | Purine nucleoside phosphorylase | -8,96 | 10 | 26 | 234 | 2 |  | 2 |  | 2 | 13 | 0,15 | ++ |
| C4XF42 | MBIO_0499 | Phosphate transport ATP-binding protein PstB | -11,19 | 15 | 29 | 256 | 2 |  | 2 |  | 2 | 13 | 0,15 | ++ |
| C4XFQ0 | MBIO_0707 | prolyl-tRNA synthetase | -15,93 | 10 | 56 | 482 | 4 |  | 4 |  | 4 | 26 | 0,15 | ++ |
| C4XEW3 | MBIO_0420 | Pyrimidine-nucleoside phosphorylase | -25,84 | 11 | 47 | 432 | 3 |  | 3 |  | 3 | 20 | 0,15 | ++ |
| C4XDW8 | MBIO_0075 | hypothetical membrane protein (1 TMD) | -13,40 | 8 | 63 | 533 | 4 |  | 4 |  | 4 | 28 | 0,14 | ++ |
| C4XEP6 | MBIO_0353 | hypothetical protein | -8,97 | 9 | 47 | 412 | 3 |  | 3 |  | 3 | 21 | 0,14 | ++ |
| C4XG44 | MBIO_0851 | histidyl-tRNA synthetase | -27,76 | 10 | 53 | 454 | 3 |  | 3 |  | 3 | 21 | 0,14 | ++ |
| C4XFM2 | MBIO_0679 | DNA polymerase III, beta chain | -16,48 | 9 | 42 | 369 | 3 |  | 3 |  | 3 | 22 | 0,14 | ++ |
| C4XFC8 | MBIO_0585 | RNA polymerase sigma factor RpoD | -9,60 | 5 | 57 | 489 | 2 |  | 2 |  | 2 | 15 | 0,13 | ++ |
| C4XFL4 | MBIO_0671 | Tyrosyl-tRNA synthetase | -14,16 | 6 | 49 | 431 | 2 |  | 2 |  | 2 | 15 | 0,13 | ++ |
| Q9X493 | MBIO_0766 | DNA polymerase III subunit | -8,86 | 10 | 35 | 305 | 2 |  | 2 |  | 2 | 15 | 0,13 | ++ |
| C4XEE4 | MBIO_0251 | ATP-binding helicase (1 TMD) | -36,83 | 9 | 130 | 1113 | 8 |  | 8 |  | 8 | 61 | 0,13 | ++ |
| C4XEP7 | MBIO_0354 | ABC transporter | -20,14 | 8 | 64 | 560 | 3 |  | 3 |  | 3 | 23 | 0,13 | ++ |
| C4XF15 | MBIO_0472 | Aminopeptidase C | -19,30 | 9 | 51 | 443 | 3 |  | 3 |  | 3 | 23 | 0,13 | ++ |
| C4XG17 | MBIO_0824 | hypothetical membrane protein (1 TMD) | -19,36 | 6 | 102 | 881 | 5 |  | 5 |  | 5 | 39 | 0,13 | ++ |
| C4XE35 | MBIO_0142 | oligoribonuclease A | -7,73 | 7 | 38 | 336 | 2 |  | 2 |  | 2 | 16 | 0,13 | ++ |
| C4XE79 | MBIO_0186 | Glutamyl-tRNA amidotransferase subunit A | -17,42 | 6 | 49 | 440 | 2 |  | 2 |  | 2 | 17 | 0,12 | ++ |
| C4XFI5 | MBIO_0642 | UDP glucose 4 epimerase | -16,92 | 11 | 38 | 331 | 2 |  | 2 |  | 2 | 17 | 0,12 | ++ |
| C4XFT2 | MBIO_0739 | hypothetical protein | -11,88 | 8 | 33 | 281 | 2 |  | 2 |  | 2 | 17 | 0,12 | ++ |
| C4XEY5 | MBIO_0442 | DUF2779 hypothetical protein | -17,95 | 6 | 89 | 752 | 4 |  | 4 |  | 4 | 35 | 0,11 | ++ |
| C4XE45 | MBIO_0152 | DUF4143 hypothetical protein | -5,68 | 5 | 58 | 490 | 3 |  | 3 |  | 3 | 27 | 0,11 | ++ |
| Q6GYZ8 | MBIO_0497 | Cytosol aminopeptidase PepA | -14,20 | 6 | 41 | 375 | 2 |  | 2 |  | 2 | 18 | 0,11 | ++ |
| C4XFY9 | MBIO_0796 | **DNA-directed RNA polymerase alpha subunit** | -13,56 | 7 | 38 | 339 | 2 |  | 2 |  | 2 | 18 | 0,11 | ++ |
| C4XF81 | MBIO_0538 | hypothetical membrane protein (2 TMD) | -5,18 | 4 | 53 | 445 | 2 |  | 2 |  | 2 | 19 | 0,11 | ++ |
| C4XF44 | MBIO_0501 | phosphate-binding protein PstS (1 TMD) | -12,44 | 6 | 44 | 378 | 2 |  | 2 |  | 2 | 20 | 0,10 | ++ |
| C4XG48 | MBIO_0855 | ABC transporter | -4,02 | 2 | 70 | 615 | 2 |  | 2 |  | 2 | 21 | 0,10 | ++ |
| C4XFK6 | MBIO_0663 | DNA methyltransferase | -10,70 | 6 | 59 | 505 | 2 |  | 2 |  | 2 | 23 | 0,09 | + |
| C4XF57 | MBIO_0514 | exoribonuclease RNase R | -5,12 | 6 | 84 | 727 | 3 |  | 3 |  | 3 | 36 | 0,08 | + |
| C4XEI4 | MBIO_0291 | phosphoglucomutase/phosphomannomutase | -16,95 | 4 | 116 | 1003 | 4 |  | 4 |  | 4 | 51 | 0,08 | + |
| C4XEX9 | MBIO_0436 | Arginyl-tRNA synthetase | -7,85 | 4 | 63 | 550 | 2 |  | 2 |  | 2 | 27 | 0,07 | + |
| C4XE80 | MBIO_0187 | Glutamyl-tRNA amidotransferase subunit B | -12,89 | 5 | 54 | 475 | 2 |  | 2 |  | 2 | 28 | 0,07 | + |
| C4XEH6 | MBIO_0283 | hypothetical membrane protein (2 TMD) | -8,03 | 3 | 145 | 1232 | 4 |  | 4 |  | 4 | 57 | 0,07 | + |
| C4XF59 | MBIO_0516 | Methionyl-tRNA synthetase | -11,33 | 5 | 61 | 516 | 2 |  | 2 |  | 2 | 29 | 0,07 | + |
| C4XFN1 | MBIO_0688 | FtsX domain, membrane protein (7 TMD) | -39,59 | 4 | 306 | 2684 | 8 |  | 8 |  | 8 | 117 | 0,07 | + |
| C4XG28 | MBIO_0835 | Mg(2+) transport ATPase, P-type | -6,63 | 2 | 103 | 918 | 2 |  | 2 |  | 2 | 32 | 0,06 | + |
| C4XF85 | MBIO_0542 | hypothetical protein | -12,34 | 5 | 79 | 665 | 2 |  | 2 |  | 2 | 35 | 0,06 | + |
| C4XEE0 | MBIO_0247 | hypothetical protein | -13,73 | 2 | 99 | 830 | 2 |  | 2 |  | 2 | 43 | 0,05 | + |
| C4XF83 | MBIO_0540 | DNA polymerase III, alpha subunit | -10,58 | 2 | 111 | 967 | 2 |  | 2 |  | 2 | 53 | 0,04 | + |
| C4XEN7 | MBIO_0344 | DNA-directed RNA polymerase beta' subunit | -5,10 | 1 | 170 | 1506 | 2 |  | 2 |  | 2 | 65 | 0,03 | + |

| lipoproteins and transmembrane proteins |
| --- |
| membrane associated proteins |
| cytoplasmic proteins |
| PAI semi quantitative scale: ++++: ≥1.0; +++: [0.5-1.0[; ++: [0.1-0.5[; + <0.1. |
| Proteins retrieved in EV from the three species are in bold |

|  | **membrane** | **membrane-bound** | **cytoplasmic** |
| --- | --- | --- | --- |
| ***M. fermentans* PG18^T^ EV** | **107,00** | **34** | **117** |
| **258 proteins** |  |  |  |
| **percentage** | **41,47** | **13** | **45** |


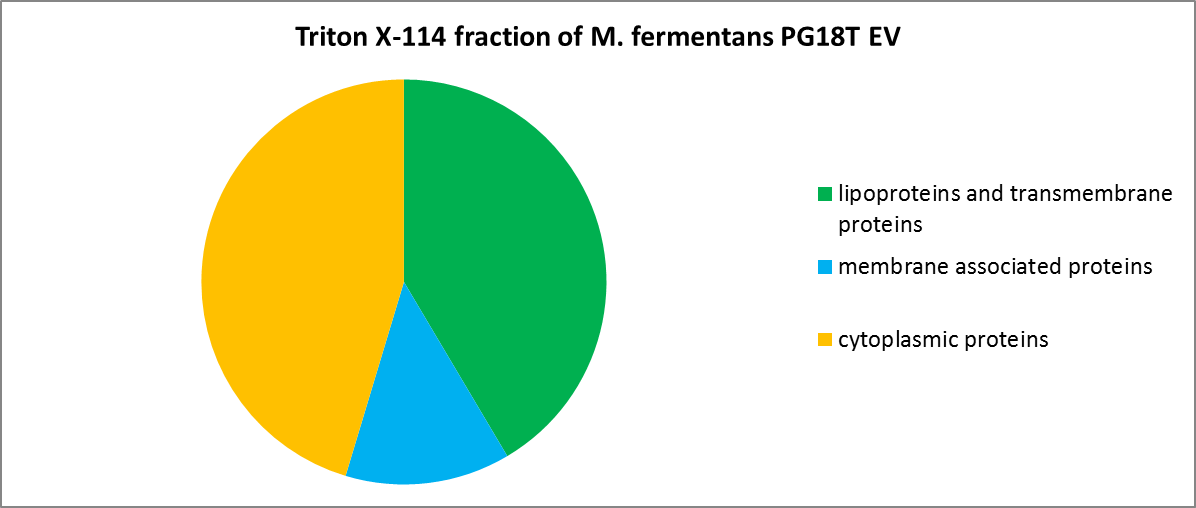

Supplement: S1 Table — Proteins are classified according to their PAI. (DOCX) [file pone.0208160.s003.docx]
